# Extending the Environmental influences on Child Health Outcomes (ECHO) Cohort through 2030: Rationale and study protocol

**DOI:** 10.1371/journal.pone.0312677

**Published:** 2024-12-26

**Authors:** Courtney K. Blackwell, David Cella, Linda Adair, José F. Cordero, Suman R. Das, Amy J. Elliott, Alison E. Hipwell, Lisa P. Jacobson, Jenae M. Neiderhiser, Joseph B. Stanford, Rosalind J. Wright, Richard Gershon, on behalf of the ECHO Cohort Consortium

**Affiliations:** 1 Department of Medical Social Sciences, Feinberg School of Medicine, Northwestern University, Chicago, Illinois, United States of America; 2 Department of Nutrition, Gillings Schools of Global Public Health, University of North Carolina, Chapel Hill, North Carolina, United States of America; 3 Department of Epidemiology & Biostatistics, University of Georgia, Athens, Georgia, United States of America; 4 Division of Infectious Diseases, Department of Medicine, Vanderbilt University Medical Center, Nashville, Tennessee, United States of America; 5 Avera Research Institute, Sioux Falls, South Dakota, United States of America; 6 Department of Pediatrics, University of South Dakota Sanford School of Medicine, Sioux Falls, South Dakota, United States of America; 7 Department of Psychiatry, University of Pittsburgh, Pittsburgh, Pennsylvania, United States of America; 8 Department of Epidemiology, Johns Hopkins University Bloomberg School of Public Health, Baltimore, Maryland, United States of America; 9 Department of Psychology, Penn State University, State College, Pennsylvania, United States of America; 10 Department of Family and Preventive Medicine, University of Utah Spencer Fox Eccles School of Medicine, Salt Lake City, Utah, United States of America; 11 Department of Public Health, Icahn School of Medicine at Mount Sinai, New York, New York, United States of America; University of Wyoming College of Health Sciences, UNITED STATES OF AMERICA

## Abstract

Early life environmental exposures, even those experienced before conception, can shape health and disease trajectories across the lifespan. Optimizing the detection of the constellation of exposure effects on a broad range of child health outcomes across development requires considerable sample size, transdisciplinary expertise, and developmentally sensitive and dimensional measurement. To address this, the National Institutes of Health (NIH) Environmental influences on Child Health Outcomes (ECHO) Cohort Study is an observational longitudinal pediatric cohort study. In the first phase from 2016–2023, the ECHO Program built a robust platform for investigating prenatal and early life environmental exposures on child health outcomes. Now, the ECHO Program is extending longitudinal follow-up of existing ECHO participants <21 years of age and recruiting and following new pregnant participants <20 weeks gestation and their offspring through 2030. Participants will be enrolled at 72 Cohort Study Sites across all 50 US states, the District of Columbia, and Puerto Rico. Exposure assessments span the biological, chemical/physical, lifestyle, and social environment; child health outcomes focus on five broad domains: pre-, peri-, postnatal; airways; obesity; neurodevelopment; and positive health, or one’s physical, mental, and social well-being. Data and biospecimens will be collected annually through August 2030, with an expected total sample size of 60,000 children and their caregivers. The ECHO Cohort Study represents the largest national longitudinal study of children’s health in the US. Here, we describe the ECHO Cohort “Cycle 2” observational study arm and the ECHO Cohort Protocol version 3.0 (ECP v3.0), which delineates the data elements, measures, and biospecimens that all ECHO Cycle 2 Cohort Study Sites will collect and analyze.

## Introduction

Early environmental exposures profoundly shape lifespan health and disease trajectories [1]. Robust evidence from pre-clinical and clinical studies shows that preconception and prenatal exposures at levels not toxic to adults can permanently impair the developing brain and associated regulatory systems, subsequently increasing susceptibility to various diseases and developmental challenges [2–4]. Even before the child is conceived, the parental exposome—the profile of an individual’s exogenous (e.g., pollutants) and endogenous (e.g., inflammation) exposures—can impact future offspring health [5], as can the maternal exposome during pregnancy [6, 7]. Because peak neuroplasticity occurs during the first five years of life, children are especially susceptible to environmental exposures that can alter brain development [8]. As chemical and psychosocial stressors often co-occur, these factors disproportionately impact historically minoritized groups such as communities of color and those of lower socioeconomic status [9–12].

The impact of such stressors on child health is often subtle, with varying developmental expression, and can be especially hard to detect during early childhood due to rapid shifts in development [9, 13, 14]. Moreover, adverse effects of the exposome are developmentally patterned, manifesting as anatomic, physiologic, behavioral, and developmental abnormalities [15–19] and/or disruptions in normative developmental processes and maturation [14, 20]. Adverse effects impact regulatory systems and multi-organ system interactions and alter susceptibility to future exposures, even without overt pathology at birth, making it challenging to differentiate specific effects [8, 20]. Finally, effects on phenotypic development can be buffered by the child’s proximal and distal protective experiences (e.g., parental responsiveness, health system intervention) and compensatory maturation (e.g., language) [21, 22]. A variety of influential factors such as diet and nutrition [23–28]. air pollution [29–32], smoking and alcohol consumption [22, 33, 34], obesity [23, 24], and psychosocial stress [35, 36] provide opportunities for public health or clinical intervention or prevention even before conception, thus ensuring improved health outcomes for pregnant individuals and children. This situation calls for: 1) developmentally sensitive measurement from preconception through early childhood; 2) a life course approach to the characterization of potential differential health effects *across* developmental periods; [37] and 3) dimensional measurement (versus categorical binary, such as a diagnostic indicator of whether a child has or does not have a specific medical condition) that captures a broad, continuous outcome spectrum to enable identification of subtle, sub-clinical exposure effects and complex relationships between and across exposures and outcomes beyond simple diagnostic indicators [9, 22]. Moreover, optimizing detection of multiple exposure effects on a broad range of child health outcomes requires considerable sample size and transdisciplinary expertise [9, 38–40].

To address these opportunities, the National Institutes of Health (NIH) Environmental influences on Child Health Outcomes (ECHO) Program brought together existing pediatric longitudinal observational cohorts comprising more than 200 sites in the US, including Puerto Rico, to form the national ECHO Cohort [41, 42]. From September 2016 to August 2023 (ECHO “Cycle 1”), the ECHO Program built a robust data and biospecimen collection and management platform, including central specimen storage and assaying, for studying prenatal and early childhood environmental exposures on pediatric health outcomes and developed the ECHO Cohort Data Platform with data from over 60,000 children and primary caregivers [43]. In collaboration with the NIH Institutional Development Award (IDeA) Program, which funds research on populations in states with historically low NIH funding, the ECHO Program also developed the ECHO IDeA States Pediatric Clinical Trials Network (ISPCTN) to address disparities in pediatric research with an emphasis on rural and underserved infants and children living in IDeA states [44]. The ECHO Cohort and ISPCTN synergized around priority research areas, with the observational cohort study providing foundational evidence on which the ISPCTN could develop clinical intervention studies.

Building on this rich foundation, the ECHO Program is extending longitudinal follow up of ECHO participants in the observational ECHO Cohort through 2030 and is funding additional Cohort Study Sites for the recruitment and follow-up of pregnant participants. Here, we describe the ECHO Cohort “Cycle 2” observational study arm and the ECHO Cohort Protocol (ECP) v3.0, which delineates the data elements, measures, and biospecimens that all ECHO Cycle 2 Cohort Study Sites will collect for future analysis. For the purposes of the ECP v3.0, data elements refer to the core concepts to be measured; measures are the specific data collection tools to assess data elements; and biospecimens are the samples of material taken from the human body, such as blood and urine.

## Materials and methods

### Objectives

The overarching objective of the NIH ECHO Program is to enhance the health of children for generations to come by investigating prenatal and early biological, physical/chemical, lifestyle, and psychosocial exposures in relation to five primary pediatric outcome areas: pre/peri/postnatal outcomes; upper and lower airways; obesity; neurodevelopment; and positive health, which describes one’s physical, mental, and social well-being [41, 42]. To do so, ECHO requires a standardized collection of core data elements and biospecimens with psychometrically robust measures and methods to enable high-quality assessment, interpretation, modeling, and analysis of the effects of early environmental exposures on child health outcomes across a diverse participant population.

### Study design

**Fig 1:**
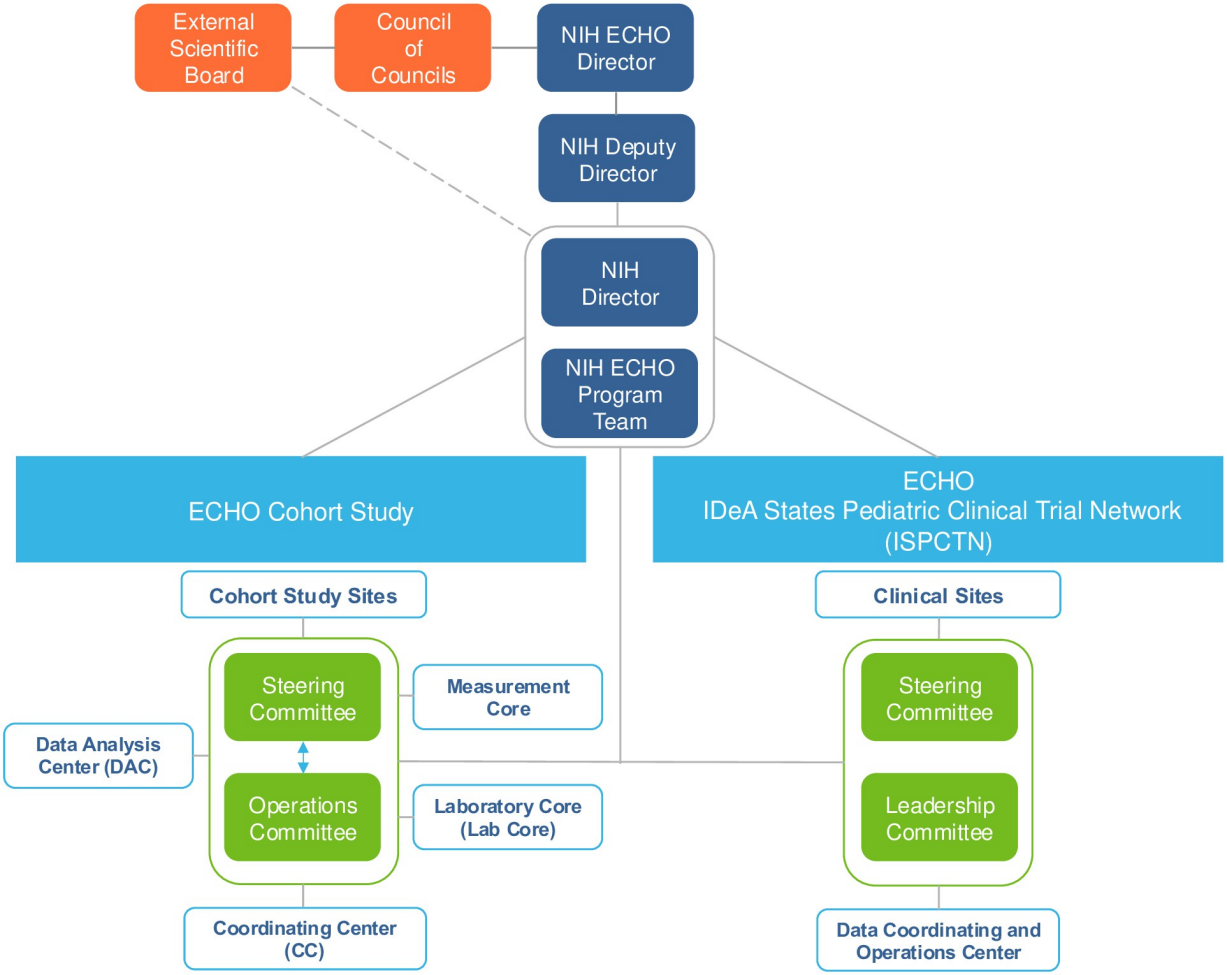
ECHO Program organizational chart. The ECHO Program is comprised of an observational study arm, the ECHO Cohort Study described in this manuscript, and the ISPCTN clinical trials arm.

The ECHO Cohort Study, funded by the NIH Office of the Director, is a longitudinal prospective study. In Cycle 2 (2023–2030), data and biospecimens will be collected from participants recruited in Cycle 1 [43] and *de novo* pregnancy participants to establish the combined ECHO Cohort. The ECHO Cycle 2 funding period started in September 2023 and includes 72 Cohort Study Sites enrolling participants, along with four Cores and Centers that help facilitate all aspects of the study, from protocol development and training to data analysis and dissemination. The four cores and centers are the Coordinating Center (CC) at the Duke Clinical Research Institute, the Data Analysis Center (DAC) at Johns Hopkins University and RTI International, the Laboratory Core (Lab Core) at Vanderbilt University Medical Center, and the Measurement Core at Northwestern University. Cohort Study Sites, Cores, and Centers comprise the ECHO Cohort Study. Investigators from all components are represented on the ECHO Steering Committee, which is charged with setting ECHO’s scientific research priorities and objectives, and the ECHO Operations Committee, which is responsible for ensuring the achievement of the Steering Committee’s priorities by overseeing and evaluating program-wide progress towards such objectives (see Fig 1 for organizational structure). The Steering and Operations Committees are in close contact with each other to ensure the program prioritizes scientific initiatives that are both important contributions to the broader field while also feasible and achievable for a large-scale consortium such as ECHO. Building from Cycle 1, the ECHO Cohort Study will leverage transdisciplinary team science to conduct solution-oriented observational longitudinal research on such topics as early origins of health disparities, identification of early critical exposure periods impacting health and development trajectories, biological pathways of risk and resiliency, and novel investigation of genetic, social, and behavioral factors that impact offspring health even before conception. Cycle 2 protocol implementation, including enrollment of and data collection from eligible persons (i.e., pregnant individuals <20 weeks gestation and Cycle 1 participants <21 years of age) began in January 2024. Data and biospecimens will be collected from each participant at least once per year through August 2030.

### Protocol development

**Fig 2:**
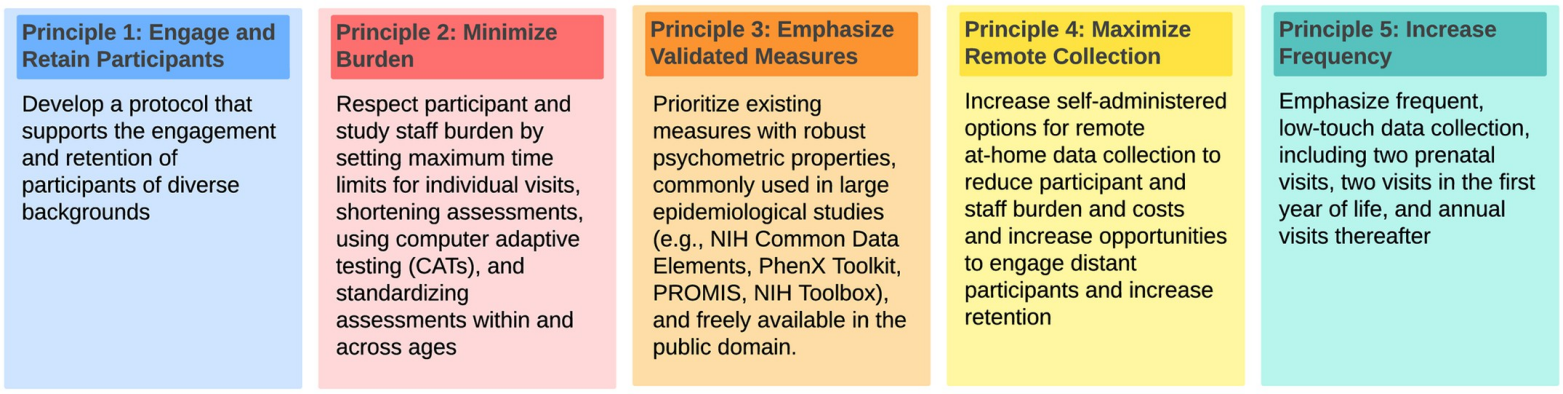
Guiding principles for protocol development. The ECHO Protocol Task Force began by establishing a set of principles to guide protocol development.

In the sixth year of Cycle 1 in January 2022, the ECHO Cycle 1 Executive Committee convened the Protocol Task Force (PTF) to develop a standard protocol for evaluating early environmental exposures on child health outcomes that balanced participant and staff burden with scientific value for use in NIH-funded prospective longitudinal cohort studies. The PTF established guiding principles (Fig 2) and a standard data collection framework that identified key exposure and outcome areas based on the Cycle 1 ECHO-Wide Cohort Protocol v2.1 [43]. The group reviewed Cycle 1 metadata (e.g., frequency of collection, item missingness, average completion time) to identify form usage rates and participant time burden per assessment to identify particularly burdensome measures and biospecimen collections to inform whether modifications or removal were required for Cycle 2 to decrease participant burden. PTF subgroups were assigned to scientific domains, and each subgroup reviewed relevant sections of the v2.1 protocol, along with the metadata and feedback from the Cycle 1 Diversity, Equity, and Inclusion (DEI) Working Group’s extensive review of data collection forms and processes, to suggest data elements and associated measures and biospecimens to include for ECP v3.0. In May 2022, the PTF met in person to collate this input and developed an initial protocol draft, paying particular attention to the concurrent collection of conceptually aligned data elements and biospecimens to allow for meaningful, scientifically robust analyses. The preliminary draft protocol underwent review and comment in July 2022 by the ECHO Executive Committee and DEI Working Group and was approved by the NIH ECHO Program Office in August 2022.

**Fig 3:**
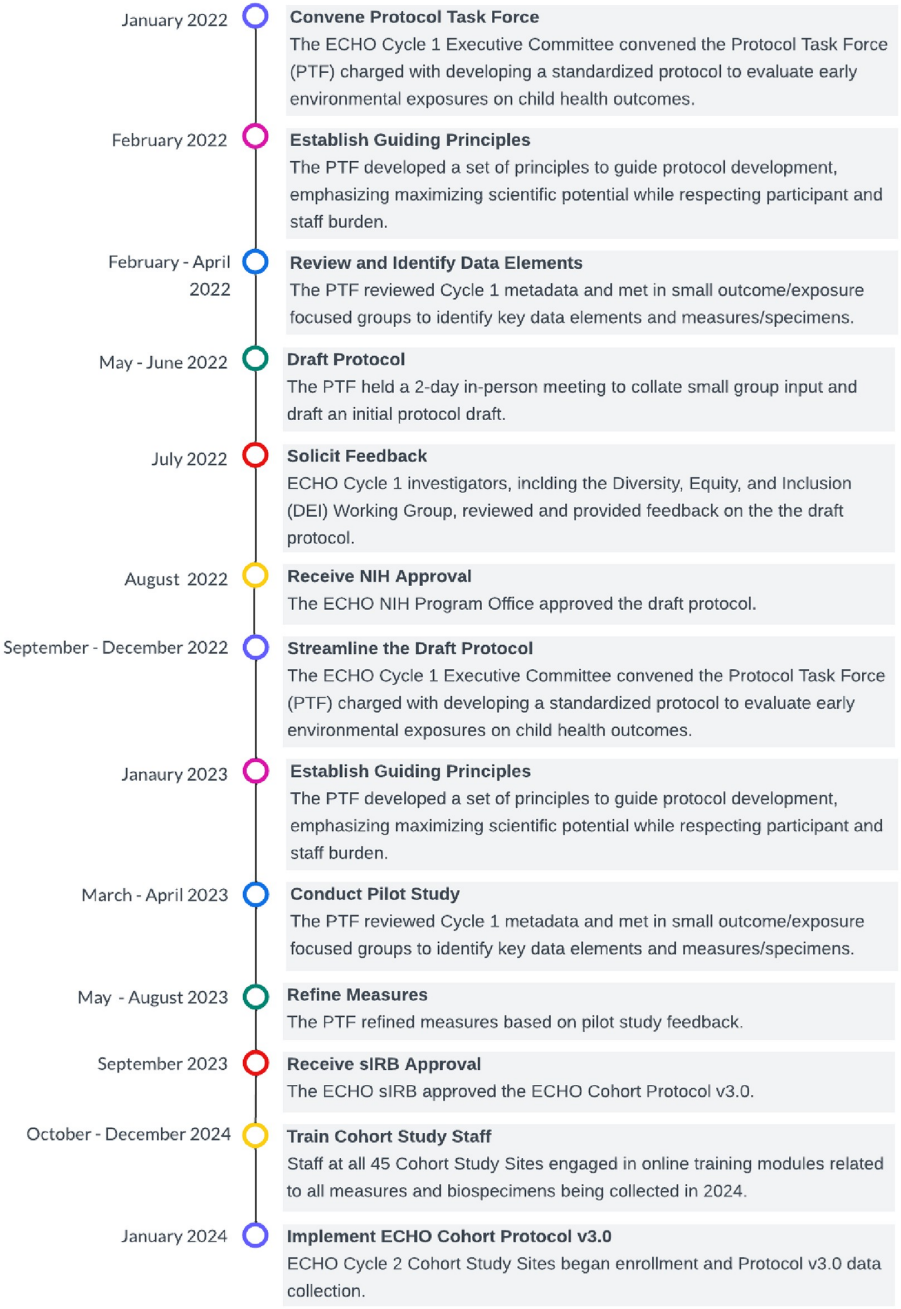
ECHO Cohort Protocol v3.0 development and implementation timeline. The ECHO Protocol Task Force developed Protocol v3.0 over 19 months, followed by three months of training and culminating in site activation and enrollment in January 2024.

In response to ECHO investigator concerns over participant and staff burden, the PTF further streamlined the protocol using three approaches: (1) identify and remove highly specific data elements, measures, and biospecimens primarily relevant to only one outcome or exposure area (e.g., measures of ADHD, skin folds, nasal swabs, breastmilk); (2) replace lengthy measures with briefer options, including computer adaptive tests and short forms; and (3) reduce data collection frequency. The PTF incorporated the first approach because subsequent protocol versions will include similar specialized elements to be administered by subsets of Cohort Study Sites with particular interest in those scientific areas. Additionally, a priority for informing the streamlining process was ensuring that most measures and biospecimens could be captured remotely by the participant or with the help of caregivers for younger children. NIH approved this second draft of ECP v3.0 in January 2023. In Spring 2023, the PTF initiated internal pilot testing of new and heavily modified data collection instruments to obtain qualitative feedback from non-ECHO participants (e.g., study staff, colleagues) on form length and comprehensibility to inform instrument modifications. The Western Copernicus Group (WCG) IRB, the study’s single IRB (sIRB), approved the final ECP v3.0 in September 2023 for implementation in January 2024. See Fig 3 for an overview of the protocol development timeline.

### Study setting and participating sites

The ECHO Cycle 2 Cohort Study comprises 45 Cohort Study Site awards, including 14 new pregnancy Site awards, 16 Site awards re-enrolling their Cycle 1 participants (children and caregivers), and 15 continuing Site awards enrolling both new pregnant individuals and re-enrolling Cycle 1 participants. Reflecting the residence of Cycle 1 participants [43], we anticipate that Cycle 2 participants will reside across all 50 US States, the District of Columbia, and Puerto Rico. Participants from ECHO Cohort Study Sites who provide informed consent and assent, as appropriate, are eligible for inclusion in the ECHO Cohort Protocol. Each Site may have individual inclusion/exclusion criteria based on the parent study [43]. These sites are heterogeneous with respect to environment, population demographics, selection criteria, and study design (for site-level descriptions, see: https://echochildren.org/echo-cohort/). A primary emphasis of participant enrollment and retention is maintaining a diverse ECHO Cohort participant population throughout the study.

**Fig 4:**
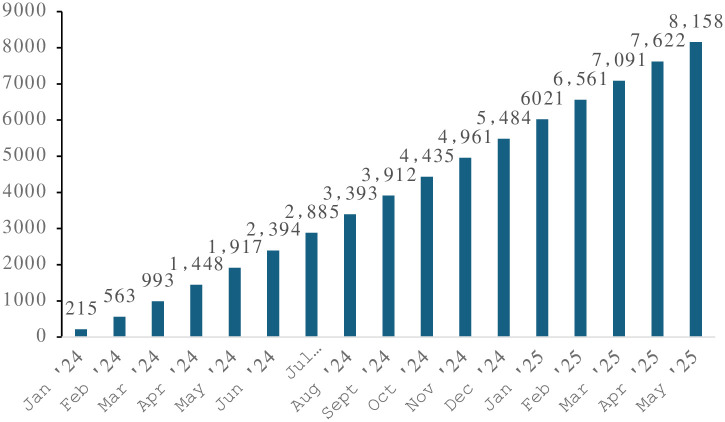
Expected pregnant person enrollment accrual by the end of each month from January 2024 to May 2025. A total of n = 8,158 pregnant people are expected to be enrolled during the UG3 phase of the ECHO Cohort Study (January 2024 –May 2025), including n = 4,649 pregnant people who self-identify as a race/ethnicity other than non-Hispanic White. By the end of ECHO Cycle 2, n = 20,000 pregnant people are expected to be enrolled.

**Fig 5:**
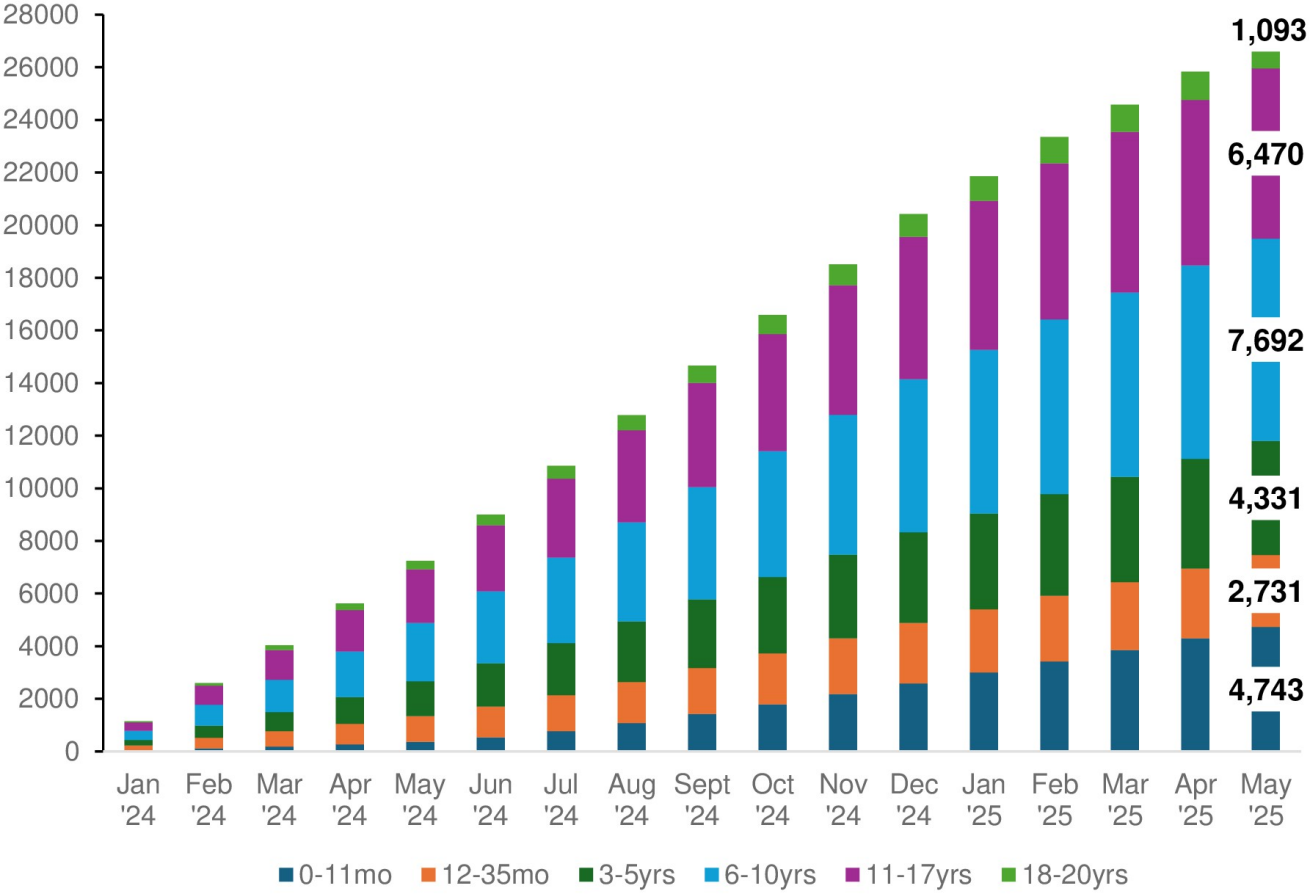
Expected child enrollment accrual by the end of each month from January 2024 to May 2025 by age band. “Age band” refers to the developmentally informed age groups of children, where all children within an age band are administered the same set of instruments and specimens within a given calendar year. A total of n = 27,050 children ages 0 to <21 years are expected to be enrolled during the UG3 phase of the ECHO Cohort Study (January 2024 –May 2025), including expected births from new pregnancies, and includes n = 11,650 children who are caregiver or self-identified as a race/ethnicity other than non-Hispanic White.

Figs 4 and 5 display the expected Cycle 2 enrollment of pregnant individuals and children, respectively, from January 5, 2024, to May 31, 2025, which reflects the initial 2-year run-in phase of Cycle 2 aligned to the UG3 phase of the NIH UG3/UH3 funding mechanism of the study. The UG3 phase focuses on the recruitment and consent of new pregnant participants, follow-up and reconsent of Cycle 1 participants, implementation of the Cycle 2 protocol, and development and dissemination of ECHO science. The NIH will consider successful UG3 Cohort Study Sites for transition to the UH3 phase based on standard criteria outlined in the original Request for Applications [45]. Enrollment is expected to continue during the UH3 phase, with approximately 20,000 pregnant individuals and 40,000 Cycle 1 children and their caregivers enrolled in Cycle 2. Additionally, Cycle 2 of the ECHO Program will include a preconception pilot study derived from the postpartum period of the 20,000 pregnant participants, with an expected 10,000 preconception participants and, when available, their partners, resulting in at least 3,000 live births with information on preconception exposures collected prospectively from the biological parents (see *Planned protocol updates* below). Combined, these strategies are expected to result in sites collecting data from approximately 60,000 children and their caregivers over the next seven years.

### Schedule of assessments

All Cohort Study Sites will collect and contribute data related to ECHO’s five primary outcomes and three primary types of exposures (described in detail below). The ECHO Program expects that each Cohort Study Site will collect measures and biospecimens on their cohort participants twice in the prenatal period (an “early” pregnancy visit before 20 weeks’ gestation and in the 3^rd^ trimester), in the perinatal period (cord blood and also placenta, when possible), and when the index child is 0 to 5 months, 6 to 11 months, 12 to 23 months, and 24 to 35 months. Beginning at age 3 years, the protocol follows a calendar-based annual visit schedule delimited into four developmentally aligned child age bands: 3 to < 6 years, 6 to < 11 years, 11 to < 18 years, and 18 to < 21 years. Age bands were selected to reflect important developmental life stages based on brain development [46], child development theory [47, 48], major national and international health organizations [49–53], with the acknowledgment that age bands provide a useful heuristic for assessment but that development is continuous, varies across individuals, and that age is a convenient way to define pediatric life stages but only one characteristic that delineates development.

**Fig 6:**
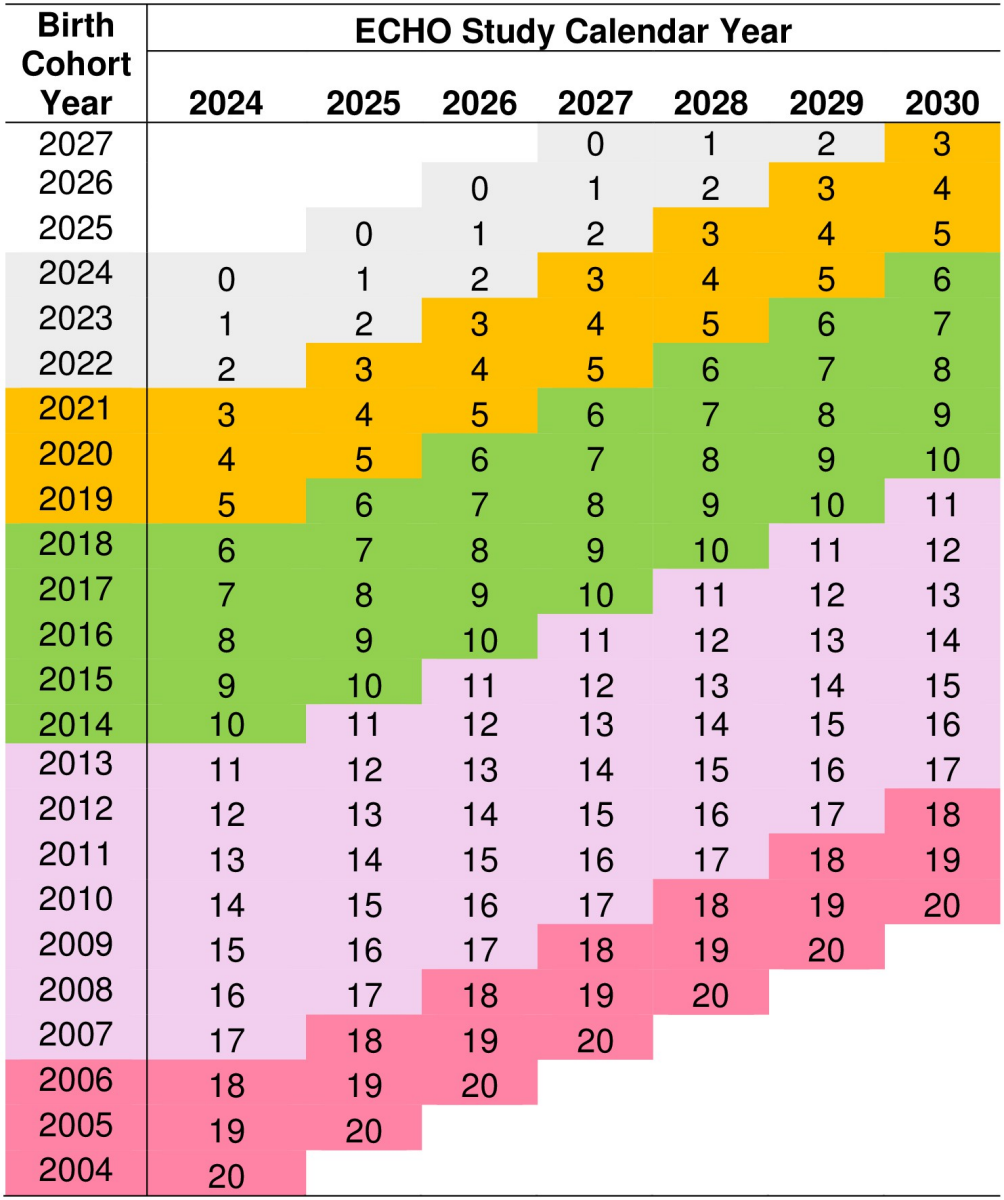
Graphical depiction of how each birth cohort progresses through age bands, by year they enter the Cycle 2 ECHO Cohort Protocol (ECP) v3.0. Birth cohort describes the group of children who were born in the same year (“Birth Cohort Year”) and enter the ECP v3.0 at the same age in the same “ECHO Study Calendar Year.” Values represent the child age in the given calendar year for the respective birth cohort. Shaded colors represent the ECP v3.0 age bands, with gray representing children ages 0 to < 36 months.

Fig 6 shows the progression of the series of birth cohorts within the larger ECHO Cohort by age band from 2024 to 2030. “Birth cohort” describes the group of children who were born in the same year and enter the ECP v3.0 at the same age in the same ECHO Study Calendar Year.

The novel ECHO age-band calendar approach means each birth cohort, those children born in the same calendar year, within the larger ECHO Cohort is administered the same set of data elements, but the same age children, born in the following calendar year and thus reflecting the next birth cohort, are administered a slightly different set of data elements. The alternative design of an age-specific protocol that is characteristic of traditional cohort studies would mean all children of a discrete age are administered the same data elements, regardless of whether children are born in the same year or were born in different years [54]. For example, a traditional cohort study recruiting pregnant participants over three years would expect to have children enter the study across multiple years, but the same set of data elements would be administered to all 1-year-olds, regardless of whether the child turned one in the second year of the study or the third year of the study. Because of the level of detail in and volume of assessments and associated participant and staff burden, this alternative age-based approach results in (1) fewer and less frequent collection of each measure and biospecimen and (2) assessment only at discreet ages versus across multiple ages, thus leaving large age gaps in when measures and biospecimens are assessed. The ECHO age-band calendar approach allows for the coverage of all data elements across all ages and is more efficient in reducing participant and staff burden–particularly for sites collecting data across a wide participant age range. This benefit is hypothesized to outweigh the complexity of children of the same age having different data collection protocols each year, depending on what year they enter the age band.

During a single calendar year (e.g., January 1 to December 31, 2024), Cohort Study Sites will collect the same set of data and biospecimens on all participants within a particular age band (e.g., any participant aged 3, 4, or 5 years in 2024 follows the same data and biospecimen collection procedures for 2024). Although each child will have annual visits, which is more frequent than prescribed in Cycle 1 when sites were required to collect measures once within an age band, not every measure or biospecimen is collected at each annual visit. Instead, measures are spread across calendar years to limit participant burden while obtaining longitudinal measurements within and across age bands. With a few exceptions of the same data elements collected annually (e.g., residential address, child medical history), data elements and biospecimens are generally collected every two to three years, resulting in two or three repeated assessments for every measure or biospecimen for all participants over the developmental lifespan. For example, caregivers of all 3-5-year-olds in 2024 will complete the *Child Behavior Checklist (CBCL) for Ages 1*.*5–5*, but in 2025, no caregivers of 3- to 5-year-olds will complete this measure. Thus, a 3-year-old in 2024 will have CBCL data, but a 3-year-old in 2025 will not. In 2026, the CBCL Ages 1.5–5 is collected again on all 3- to 5-year-olds. Thus, the group of 3-year-olds in 2024 will have CBCL data when they are 3 and 5 years old, while the group of 3-year-olds in 2025 will have CBCL data when they are 4 years old. The Protocol Task Force acknowledged this method may complicate longitudinal analyses as not all participants will have every measure at the same age–only within the same age band; a subset of participants will have any one specific measure at any one specific age. However, the alternative age-based protocol would result in similar complexity, with large gaps in ages for individual measures that complicate longitudinal analyses in other ways. For example, if the CBCL Ages 1.5–5 was collected on all 3- and 5-year-olds, there would be no CBCL data on 4-year-olds, one time point of data for 5-year-olds, and two time points for 3-year-olds. While all 3- and 5-year-olds would be administered the measure, only longitudinal data would exist for those who were 3 or younger when enrolled in ECHO Cycle 2. Moreover, this age-based protocol completely skips certain ages, when important developmental changes occur. The age-band calendar approach enables all ages within an age band to be captured. With the inability to collect all measures on all children in all years of the study, there was no way to avoid such complexity.

### Assessments

The ECP v3.0 delineates data elements and associated measures (e.g., surveys, physical measurements) and biospecimens for collection from pregnant individuals and children ages 0 to <21 years. In addition to the sociodemographic and health history assessments of pregnant people, children, and families (Table 1), the protocol focuses on ECHO’s three primary exposure areas and five primary child health outcomes. An overview of data elements and associated measures and biospecimens is provided below, and complete details regarding administration timing and procedures and copies of all data collection forms are available at ECHOChildren.org.

**Table 1:** Data elements and measures to assess sociodemographic characteristics and medical history^a^^,^^b^.

Data Element	Measure	Prenatal	0-35mo	2024	2025	2026	2027	2028	2029	2030
**Sociodemographic Characteristics**										
Family Demographics	Demographics of the Biological Family Questionnaire^c^	Assess at first point of contact with participants following consent and enrollment								
Family Demographics	Demographics of Primary Caregiver Family Questionnaire^c^	Assess at first point of contact with participants following consent and enrollment								
Child Demographics	Demographics of the Child Questionnaire	Assess at first point of contact with participants following consent and enrollment								
Residential Address	Residential Address History Questionnaire	Assess at first point of contact with participants following consent and enrollment								
Updated Residential Address	Residential Address Update Questionnaire	Early, 3^rd^ tri	0-5mo, 6-11mo, 12-23mo, 24-35mo	3-5yr, 6-10yr, 11-17yr, 18-20yr	3-5yr, 6-10yr, 11-17yr, 18-20yr	3-5yr, 6-10yr, 11-17yr, 18-20yr	3-5yr, 6-10yr, 11-17yr, 18-20yr	3-5yr, 6-10yr, 11-17yr, 18-20yr	3-5yr, 6-10yr, 11-17yr, 18-20yr	3-5yr, 6-10yr, 11-17yr, 18-20yr
Household Income, Receipt of Federal Assistance, Financial Strain	Income, Assistance, and Financial Strain—Pregnancy Questionnaire	Early								
Household Income, Receipt of Federal Assistance, Financial Strain	Income, Assistance, and Financial Strain—Childhood Questionnaire		6-11mo	3-5yr, 6-10yr, 11-17yr			3-5yr, 6-10yr, 11-17yr			3-5yr, 6-10yr, 11-17yr
Household Income, Receipt of Federal Assistance, Financial Strain	Income, Assistance, and Financial Strain—18–20 yr olds Questionnaire			18-20yr			18-20yr			18-20yr
Caregiver & Partner Occupation	Caregiver Occupation and Employment Questionnaire	3^rd^ tri	6-11mo	3-5yr, 6-10yr, 11-17yr			3-5yr, 6-10yr, 11-17yr			3-5yr, 6-10yr, 11-17yr
Household Primary Language Spoken, Read	Language and Acculturation—Pregnancy Questionnaire	Early								
Household Primary Language Spoken, Read	Language and Acculturation—Childhood Questionnaire		12-23mo	3-5yr, 6-10yr, 11-17yr			3-5yr, 6-10yr, 11-17yr			3-5yr, 6-10yr, 11-17yr
Household Primary Language Spoken, Read	Language and Acculturation—18–20 yr olds Questionnaire			18-20yr			18-20yr			18-20yr
Early Care and Education	Early Care and Education Questionnaire		6-11mo, 12-23mo	3-5yr			3-5yr			3-5yr
K12 Education	Child K12 Education Questionnaire			6-10yr, 11-17yr			6-10yr, 11-17yr			6-10yr, 11-17yr
Gender Identity	Gender Identity—Parent Report Questionnaire			6-10yr			6-10yr			
Gender Identity	Gender Identity—Child Self-Report Questionnaire			11-17yr, 18-20yr			18-20yr			11-17yr, 18-20yr
**Child and Family Medical History**										
Child Medical History	Medical History of the Child Questionnaire		Assess at first point of contact with participants following consent and enrollment							
Child Medical History	Medical History of the Child Update Questionnaire		6-11mo, 12-23mo, 24-35mo	3-5yr, 6-10yr, 11-17yr	3-5yr, 6-10yr, 11-17yr	3-5yr, 6-10yr, 11-17yr	3-5yr, 6-10yr, 11-17yr	3-5yr, 6-10yr, 11-17yr	3-5yr, 6-10yr, 11-17yr	3-5yr, 6-10yr, 11-17yr
Child Medical History	Medical History of the Child Update—18–20 yr olds Questionnaire			18-20yr	18-20yr	18-20yr	18-20yr	18-20yr	18-20yr	18-20yr
Lifetime/Updated Family Medical Conditions	Medical History of the Family Questionnaire	Early	0-5mo, 12-23mo, 24-35mo	3-5yr, 6-10yr, 11-17yr, 18-20yr	3-5yr, 6-10yr, 11-17yr, 18-20yr	3-5yr, 6-10yr, 11-17yr, 18-20yr	3-5yr, 6-10yr, 11-17yr, 18-20yr	3-5yr, 6-10yr, 11-17yr, 18-20yr	3-5yr, 6-10yr, 11-17yr, 18-20yr	3-5yr, 6-10yr, 11-17yr, 18-20yr
Caregiver and Child Health Insurance Status	Health Insurance Coverage Questionnaire	Early	6-11mo	3-5yr, 6-10yr, 11-17yr			3-5yr, 6-10yr, 11-17yr			3-5yr, 6-10yr, 11-17yr
Caregiver and Child Health Insurance Status	Health Insurance Coverage—18–20 yr olds Questionnaire			18-20yr			18-20yr			18-20yr
Caregiver Health Literacy	Caregiver Health Literacy Questionnaire		0-5mo							

Abbreviations: mo = month; yr = year; 3^rd^ tri = 3^rd^ trimester; early = early pregnancy visit < 20 weeks gestation.

^a^The full ECHO Cohort Protocol is available at ECHOChildren.org.

^b^See S1 Table for a list of relevant measure references.

^c^Cohort Study Sites are expected to collect demographic information related to the ECHO child’s biological family members; however, in cases where biological family members are not the primary caregivers of the child (e.g., child is adopted, biological caregivers deceased), this information may not be available. In these cases, capturing the child’s rearing family demographics is critical. If the rearing family is different than the biological family, ECHO cohorts should strive to collect both biological family and rearing family demographics to the extent possibl

Table 2 details the pregnancy-related assessments and three environmental exposure areas: physical/chemical, lifestyle, and psychosocial. Surveys administered to the pregnant person or caregiver constitute the primary data collection method in these domains; exceptions include direct assessment of pregnant persons’ anthropometry and caregiver cognition, wearable technology for pregnant persons’ physical activity and sleep, and abstraction of prenatal and birth medical records.

**Table 2:** Data elements and measures to assess exposures by calendar year and age band^a^^,^^b^.

Data Element	Measure	Prenatal	0-35mo	2024	2025	2026	2027	2028	2029	2030
**Pregnancy-related**										
Maternal Height and Weight	Pregnancy Anthropometry	Early, 3rd tri	0-5mo							
Pregnancy Medical Record Abstraction	Maternal Medical Record Abstraction		6-11mo							
Maternal Supplements in Pregnancy/ Breastfeeding	Maternal Supplements Short Form Questionnaire	3rd tri	0-5mo							
Maternal Sleep Health in Pregnancy	Sleep Health in Pregnancy Questionnaire	Early, 3rd tri								
Physical Activity and Sleep in Pregnancy	Wearable activity tracker and Sleep/Physical Activity Diary	Early								
Maternal food Contaminants in Pregnancy	Maternal Food Source and Preparation Questionnaire	Early								
Maternal Diet in Pregnancy	Diet History Questionnaire (DHQ) III	Early								
Maternal Diet in Pregnancy	NHANES Dietary Screener Questionnaire	3rd tri								
**Chemical/Physical Exposures**										
Chemical Exposures	Household Chemical Exposures Questionnaire	3rd tri	12-23mo		3-5yr			3-5yr		3-5yr
Household Exposure to Secondhand Smoke	Household Exposure to Secondhand Smoke Questionnaire	3rd tri	0-5mo, 12-23mo	18-20yr	3-5yr, 6-10yr, 11-17yr		18-20yr	3-5yr, 6-10yr, 11-17yr		3-5yr, 6-10yr, 11-17yr, 18-20yr
**Lifestyle Exposures**										
Infant feeding and introduction to solids	Infant Feeding Practices Questionnaire		0-5mo, 6-11mo, 12-23mo							
Child Diet	NHANES Dietary Screener Questionnaire				3-5yr, 6-10yr, 11-17yr, 18-20yr		6-10yr, 11-17yr, 18-20yr	3-5yr	6-10yr, 11-17yr, 18-20yr	3-5yr
Child Sleep Health	Sleep Health Questionnaires		0-5mo, 6-11mo, 12-23mo, 24-35mo		3-5yr, 6-10yr, 11-17yr, 18-20yr		3-5yr, 6-10yr, 11-17yr, 18-20yr		6-10yr, 11-17yr, 18-20yr	3-5yr
Child Risk Behaviors	Youth Risk Behavior—Substance Use and Sexual Behavior Questionnaires			18-20yr		11-17yr, 18-20yr		11-17yr, 18-20yr		11-17yr, 18-20yr
Endurance/Fitness	NIH Toolbox 2-minute walk test V3*				6-10yr, 11-17yr, 18-20yr		18-20yr		6-10yr, 11-17yr, 18-20yr	
Child Device-Measured Physical Activity, Sleep	Wearable activity tracker and Sleep/Physical Activity Diary				3-5yr, 6-10yr, 11-17yr, 18-20yr		18-20yr	3-5yr	6-10yr, 11-17yr, 18-20yr	3-5yr
Home Screen Media Access & Use	Child Media Use		12-23mo	6-10yr, 11-17yr	3-5yr, 18-20yr		3-5yr	11-17yr, 18-20yr	6-10yr	3-5yr
**Psychosocial Exposures**										
Caregiver Stressful Life Events	Life Stressor Checklist—Revised (LSC-R)	Early								
Caregiver Stressful Life Events	Crisis in the Family Systems—Revised (CRISYS-R) Short Form	3rd tri	12-23mo	3-5yr, 6-10yr, 11-17yr			3-5yr, 6-10yr, 11-17yr			3-5yr, 6-10yr, 11-17yr
Caregiver Stressful Life Events	PRAMS Stressful Life Events in Pregnancy Questionnaire		0-5mo							
Caregiver Discrimination	The Everyday Discrimination Scale	3rd tri	0-5mo, 12-23mo	3-5yr, 6-10yr, 11-17yr, 18-20yr			3-5yr, 6-10yr, 11-17yr, 18-20yr			3-5yr, 6-10yr, 11-17yr
Caregiver Depressive Symptoms	PROMIS v1.0—Depression 8a/CAT	3rd tri	0-5mo, 12-23mo	3-5yr, 6-10yr, 11-17yr, 18-20yr			3-5yr, 6-10yr, 11-17yr, 18-20yr			3-5yr, 6-10yr, 11-17yr
Caregiver Anxiety	PROMIS v1.0—Anxiety 8a/CAT	3rd tri	0-5mo, 12-23mo	3-5yr, 6-10yr, 11-17yr, 18-20yr			3-5yr, 6-10yr, 11-17yr, 18-20yr			3-5yr, 6-10yr, 11-17yr
Caregiver Perceived Stress	NIH Toolbox v2.0—Perceived Stress Scale 10-Item	3rd tri	0-5mo, 12-23mo	3-5yr, 6-10yr, 11-17yr, 18-20yr			3-5yr, 6-10yr, 11-17yr, 18-20yr			3-5yr, 6-10yr, 11-17yr
Caregiver Social Support	PROMIS v2.0—Emotional Support 4a/CAT, Informational Support 4a/CAT, Instrumental Support 4a/CAT	3rd tri	0-5mo, 12-23mo	3-5yr, 6-10yr, 11-17yr, 18-20yr			3-5yr, 6-10yr, 11-17yr, 18-20yr			3-5yr, 6-10yr, 11-17yr
Caregiver Cognitive Functioning	NIH Mobile Toolbox Cognition Battery V3					3-5yr, 6-10yr, 11-17yr, 18-20yr			3-5yr	
Caregiver Global Health	PROMIS v1.2—Global Health Scale	3rd tri	6-11mo, 24-35mo	3-5yr			3-5yr			3-5yr
Caregiver Life Satisfaction	PROMIS v1.0—General Life Satisfaction 5a/CAT		0-5mo	3-5yr			3-5yr			3-5yr
Caregiver Sleep Health	Sleep Health of Adults		0-5mo, 12-23mo	3-5yr			3-5yr			3-5yr
Neighborhood Capital	Neighborhood Collective Efficacy Questionnaire		12-23mo	3-5yr, 6-10yr, 11-17yr			3-5yr		6-10yr, 11-17yr	
Caregiver-Partner Relationship Quality	Couples Satisfaction Index—4-item short form	Early	0-5mo, 12-23mo	3-5yr, 6-10yr, 11-17yr			3-5yr, 6-10yr, 11-17yr		6-10yr	3-5yr
Caregiving Quality & Behavior	Alabama Parenting Questionnaires		12-23mo	3-5yr, 11-17yr	6-10yr	11-17yr	3-5yr, 6-10yr	11-17yr		3-5yr, 6-10yr, 11-17yr
Family Relationships	PROMIS v1.0—Family Relationships 4a—Early Childhood, Parent Proxy/CAT, Pediatric/CAT		12-23mo	3-5yr, 11-17yr	6-10yr	11-17yr	3-5yr, 6-10yr	11-17yr		3-5yr, 6-10yr, 11-17yr
Peer Relationships	PROMIS v1.0—Peer Relationships 4a—Early Childhood Parent Report / PROMIS v2.0—Peer Relationships 7a—Parent Proxy/CAT / PROMIS v2.0—Peer Relationships 8a—Pediatric/CAT			3-5yr, 11-17yr	6-10yr	11-17yr	3-5yr, 6-10yr	11-17yr		3-5yr, 6-10yr, 11-17yr
Peer Relationships	PROMIS v2.0—Companionship 6a			18-20yr		18-20yr		18-20yr		18-20yr
Social Support	NIH Toolbox v2.0—Emotional Support/CAT			11-17yr, 18-20yr		11-17yr, 18-20yr		11-17yr, 18-20yr		11-17yr, 18-20yr
Bullying/Victimization	Healthy Pathways Bullying Scales			11-17yr		11-17yr		11-17yr		11-17yr
Child Negative Life Events	Adverse Childhood Experiences Questionnaire				3-5yr, 6-10yr, 11-17yr, 18-20yr		3-5yr, 6-10yr, 11-17yr, 18-20yr			3-5yr, 6-10yr, 11-17yr, 18-20yr
**Biospecimens** ^c^										
Child Specimens	Saliva (for DNA)				3-5yr		3-5yr			3-5yr
Child Specimens	Hair		12-23mo		3-5yr, 6-10yr, 11-17yr		3-5yr		6-10yr, 11-17yr	
Child Specimens	Urine		0-5mo		3-5yr, 6-10yr, 11-17yr		3-5yr		6-10yr, 11-17yr	
Child Specimens	Blood Spot		0-5mo, 12-23mo	18-20yr	3-5yr		3-5yr, 18-20yr		18-20yr	3-5yr
Child Specimens	Whole Blood*				6-10yr, 11-17yr				6-10yr, 11-17yr	
Child Specimens	Shed Teeth			6-10yr	6-10yr	6-10yr	6-10yr	6-10yr	6-10yr	6-10yr
Child Specimens	Stool		0-5mo							
Pregnant Person Specimens	Whole Blood^d^*	Early, 3rd tri								
Pregnant Person Specimens	Hair		0-5mo							
Pregnant Person Specimens	Urine	Early, 3rd tri								
Pregnant Person Specimens	Placenta		At birth							
Pregnant Person Specimens	Cord Blood		At birth							

Abbreviations: mo = month; yr = year; 3^rd^ tri = 3^rd^ trimester; early = early pregnancy visit < 20 weeks gestation.

*Denotes the data element or specimen must be collected in-person.

^a^The full ECHO Cohort Protocol is available at ECHOChildren.org.

^b^See S1 Table for a list of relevant measure references.

^c^All biospecimens except whole blood, placenta, and cord blood collection have at-home collection options in addition to in-person researcher-administered collection procedures.

^d^If the pregnant person refuses whole blood at both the early pregnancy and 3^rd^ trimester visits, Cohorts Study Sites should try to obtain a saliva sample at the 3^rd^ trimester or 0–5 month visit.

Table 3 outlines outcome assessments by the five primary ECHO outcome domains (pre-, peri-, postnatal, airways, obesity, neurodevelopment, and positive health). Instruments include caregiver-report surveys across all child ages, child self-report surveys beginning at age 8, direct assessment of child cognition and physical measures (anthropometry, blood pressure), and wearable technology for collecting child physical activity and sleep patterns.

**Table 3:** Data elements and measures to assess ECHO’s five primary child health outcomes by calendar year and age band^a^^,^^b^.

Data Element	Measure	0-35mo	2024	2025	2026	2027	2028	2029	2030
**Pre-, Peri-, Postnatal**									
Birth Outcomes	Childbirth / Neonatal Medical Record Abstraction	0-5mo							
Birth Outcomes	Child Birth Information Questionnaire	6-11mo							
**Airways**									
Airways-related	Airways Questionnaires	6-11mo, 24-35mo		3-5yr, 6-10yr, 11-17yr	18-20yr		3-5yr, 6-10yr, 11-17yr	18-20yr	
Lung Function	Spirometry			6-10yr, 11-17yr	18-20yr		6-10yr, 11-17yr	18-20yr	
**Obesity**									
Child Anthropometry and Physical Measurement	Length/Height and Weight	0-5mo, 6-11mo, 12-23mo, 24-35mo	3-5yr, 6-10yr, 11-17yr, 18-20yr	3-5yr, 6-10yr, 11-17yr, 18-20yr	3-5yr, 6-10yr, 11-17yr, 18-20yr	3-5yr, 6-10yr, 11-17yr, 18-20yr	3-5yr, 6-10yr, 11-17yr, 18-20yr	3-5yr, 6-10yr, 11-17yr, 18-20yr	3-5yr, 6-10yr, 11-17yr, 18-20yr
Child Anthropometry and Physical Measurement	Bioimpedance		5yr, 6-10yr, 11-17yr, 18-20yr	5yr, 6-10yr, 11-17yr, 18-20yr	5yr, 6-10yr, 11-17yr, 18-20yr	5yr, 6-10yr, 11-17yr, 18-20yr	5yr, 6-10yr, 11-17yr, 18-20yr	5yr, 6-10yr, 11-17yr, 18-20yr	5yr, 6-10yr, 11-17yr, 18-20yr
Child Anthropometry and Physical Measurement	Head Circumference	0-5mo, 24-35mo							
Child Anthropometry and Physical Measurement	Waist Circumference	24-35mo	18-20yr	,	3-5yr, 6-10yr, 11-17yr	18-20yr		3-5yr	6-10yr, 11-17yr, 18-20yr
Child Anthropometry and Physical Measurement	Blood Pressure and Pulse Rate		18-20yr		3-5yr, 6-10yr, 11-17yr	18-20yr		3-5yr	6-10yr, 11-17yr, 18-20yr
Child Pubertal Development	Pubertal Development Scale		6-10yr, 11-17yr	6-10yr, 11-17yr	6-10yr, 11-17yr	6-10yr, 11-17yr	6-10yr, 11-17yr	6-10yr, 11-17yr	6-10yr, 11-17yr
**Neurodevelopment**									
Developmental Milestones	Ages & Stages Questionnaire	6-11mo, 24-35mo							
General Cognition	NIH Toolbox Cognition Battery V3^c^			3-5yr, 6-11yr, 11-17yr			3-5yr	6-11yr, 11-17yr	
General Cognition	NIH Mobile Toolbox Cognition Battery V3			18-20yr			18-20yr		
Temperament	Infant Behavior Questionnaire Revised (IBQ-R)—Very Short Form / Early Childhood Behavior Questionnaire (ECBQ)—Very Short Form / Childhood Behavior Questionnaire (CBQ)—Very Short Form	6-11mo, 24-35mo		3-5yr			3-5yr		
Autism Spectrum Disorder (ASD) Screener	Modified Checklist for Autism in Toddlers Revised (M-CHAT-R)	24-35mo							
Anxiety	PROMIS v2.0—Anxiety 8a –Pediatric/CAT / PROMIS v1.0—Anxiety 8a/CAT		11-17yr, 18-20yr	6-10yr	11-17yr	6-10yr, 18-20yr	11-17yr, 18-20yr	6-10yr	611-17yr, 18-20yr
Depressive Symptoms	PROMIS—Depressive Symptoms CAT–Pediatric / PROMIS v1.0—Depression 8a/CAT		11-17yr, 18-20yr	6-10yr	11-17yr	6-10yr, 18-20yr	11-17yr	6-10yr	11-17yr, 18-20yr
Emotional & Behavioral Functioning	Child Behavior Checklist (CBCL)—1.5-5yr/6-18yr	24-35mo	3-5yr, 6-10yr, 11-17yr		3-5yr	6-10yr, 11-17yr	3-5yr		6-10yr, 11-17yr
Social Cognition	Social Responsiveness Scale—Second Edition (SRS-2) Preschool–Short Form / School Age–Short Form		3-5yr, 11-17yr	6-10yr		3-5yr, 6-10yr, 11-17yr			6-10yr, 11-17yr
Perceived Stress	PROMIS v1.0—Psychological Stress Experiences 4a—Parent Proxy/CAT, Pediatric/CAT		11-17yr	6-10yr	11-17yr	6-10yr	11-17yr	6-10yr	11-17yr
Perceived Stress	NIH Toolbox v2.0—Perceived Stress Scale 10-Item		18-20yr			18-20yr			18-20yr
Academic Ability	Early Academic Competencies Questionnaire		3-5yr			3-5yr			3-5yr
**Positive Health**									
Global Health	PROMIS v1.0—Global Health Scale 8a—Early Childhood Parent Report / PROMIS v1.0—Global Health Scale 7+2—Parent Proxy/Pediatric / PROMIS v1.2—Global Health Scale	24-35mo	3-5yr, 11-17yr, 18-20yr	6-10yr	11-17yr	3-5yr, 6-10yr, 18-20yr	11-17yrr	6-10yr	3-5yr, 11-17yr, 18-20yr
Curiosity	PROMIS v1.0—Engagement—Curiosity 6a—Early Childhood Parent Report	24-35mo	3-5yr			3-5yr			3-5yr
Flexibility	PROMIS v1.0—Self-Regulation—Flexibility 5a—Early Childhood Parent Report	24-35mo	3-5yr			3-5yr			3-5yr
Life Satisfaction	PROMIS v1.0—Life Satisfaction 8b—Parent Proxy/CAT, Pediatric/CAT / PROMIS v1.0—General Life Satisfaction 5a/CAT		11-17yr, 18-20yr	6-10yr	11-17yr, 18-20yr	6-10yr	11-17yr, 18-20yr	6-10yr	11-17yr, 18-20yr
Meaning and Purpose	PROMIS v1.0—Meaning and Purpose 8a—Pediatric/CAT / PROMIS v1.0—Meaning and Purpose 8a/CAT		11-17yr, 18-20yr		11-17yr	18-20yr	11-17yr		11-17yr, 18-20yr

Abbreviations: mo = month; yr = year; 3^rd^ tri = 3^rd^ trimester; early = early pregnancy visit < 20 weeks gestation.

^a^The full ECHO Cohort Protocol is available at ECHOChildren.org.

^b^See S1 Table for a list of relevant measure references.

^c^The NIH Toolbox Cognition Battery V3 includes a series of direct assessments that have age-based versions. In ECHO, the NIH Toolbox Early Cognition Battery is administered to children ages 4–6 years and the Cognition Battery is administered to children ages 7–17 years. The NIH Toolbox Cognition Battery V3 can be administered remotely by study staff using the NIH Toolbox participant app, which facilitates video meetings with study staff, who observe and facilitate remote administration. Alternatively, traditional in-person administration is also available.

With few exceptions, all data—including cognition, anthropometric and physical measurements, and most biospecimens—can be collected remotely with little or no direct study staff involvement. The few data elements and biospecimens that require in-person assessment are noted in Tables 1–3.

### Data collection and management

Data are collected using a centralized data capture system, “REDCap Central,” hosted by the ECHO DAC and stored in a Federal Information Security Management Act (FISMA) moderate environment. REDCap (Research Electronic Data Capture) is a metadata-driven electronic data capture program developed by Vanderbilt University for web and smartphone use [55, 56]. Data entered into this system by the Cohort Study Site staff and participants are captured in real-time in a central, secure database at the ECHO DAC. For several measures, such as the NIH Toolbox Cognition Battery, Cohort Study Sites will use a third-party application for data collection and then upload the data to the DAC’s secure FISMA moderate platform. Biospecimens are sent to and stored centrally at the Lab Core for future analyses.

Successful implementation of the ECP v3.0 is defined as (1) Cohort Study Sites administer the measures as intended, and (2) data collected are of high quality (e.g., complete, in range). One unique feature of the ECP v3.0 is the emphasis on remote data collection, with most measures and biospecimens utilizing self-administration strategies (see measures marked with an asterisk in Tables 1–3 for exceptions). In addition to self-administered questionnaires and self-collected biospecimens, the ECP v3.0 includes at-home caregiver-measured measurements to assess child length/height, weight, bioimpedance, head and waist circumference, and blood pressure. The ECHO Cycle 1 Remote Assessment Task Force conducted a remote validation pilot study to evaluate the feasibility and validity of this remote data collection strategy and confirmed that caregivers of 0- to 17-year-olds could obtain valid physical measurements from their children using study-provided standard equipment with similar reliability to researcher-administered assessments [57]. Drawing on these findings, the PTF developed instructional manuals and equipment specifications so that ECP v3.0 could be implemented in-person by study staff or remotely by caregivers (for child assessments) or pregnant individuals (for self-assessments). Cohort Study Sites are responsible for purchasing equipment and orchestrating shipping to and from participants. Except for length/height and weight for which sites may use hospital-grade equipment (e.g., stadiometer), sites conducting in-person visits must use the same equipment specified for remote administration to ensure standardization between sites and over time.

In addition to ongoing and booster training for Cohort Study Site staff and in-person and virtual site visits by the NIH Program Office and ECHO Cores/Centers to enhance implementation fidelity, an Implementation Fidelity Task Force comprised of investigators from the ECHO Cores/Centers and Cohort Study Sites will engage in the ongoing review of metadata related to measure completeness and administration adherence (e.g., study staff administered the correct assessment to a specific participant/respondent in the proper age band) to evaluate Cohort Study Site operation alignment with the protocol-prescribed data collection procedures denoted in the ECP v3.0. One function of the REDCap Central system is the creation of a participant-specific dashboard that presents the appropriate assessments by age and calendar; this function should minimize deviations from the prescribed protocol. Ongoing metadata monitoring by the Implementation Fidelity Task Force will help identify sites that may require additional training and those with the successful implementation that can provide guidance on lessons learned and best practices to help other sites.

Maintaining a continuous quality review process is essential, particularly for new measures and those with significant changes. Even when data are collected correctly, data quality flags may indicate a need to change the data collection approach and/or the measure itself. For example, measures with high rates of missingness may indicate the need for a change, particularly if the pattern of missingness is informative (i.e., not random) and indicative of significant disparities in responses related to primary scientific aims or for unique participant subgroups. Differential rates of missing data across measures may impact the ECHO Program’s ability to test specific hypotheses, and any non-random nature of missing data may increase the likelihood of producing biased estimates. Such quality issues may stem from unclear item wording, confusing skip logic, and excessive measure length–factors that may be modifiable once identified. Automated data quality check programs will be run routinely to screen for missing data, check for illogical values, compute the maximum number of consecutive items with the same response values, identify reverse-coded item inconsistencies, and screen for disproportionate rates of floor or ceiling scores. These quality checks were conducted during Cycle 1 and informed the development and modification of Cycle 2 measures. To help better understand these potential implementation fidelity concerns, we will draw on metadata, such as age-band of administration, Cohort Study Site, mode of administration, visit choreography, completion time, and, if available/relevant, individual study staff conducting the data collection. This information will help further elucidate patterns that can be targeted for remediation.

### Harmonization and statistical analyses

The ECP v3.0 will provide opportunities to characterize the impact of a broad range of early environmental exposures on child health and development. Investigators will pose specific research questions and hypotheses through an established ECHO manuscript development process, starting with submitting an initial analysis proposal detailing the study design. Project-specific statistical methodologies will be identified and executed to fit the data structure and project aims. As such, no singular analytic strategy will be employed across all scientific initiatives. Proposals undergo review by the ECHO Publications Committee, comprised of investigators from across the ECHO network (Sites, Cores, and Centers) who provide feedback early in the analysis process to help establish scientific and methodological rigor prior to the commencement of data analysis.

Cycle 1 versions of the ECHO protocol allowed for more variability in data collection measures and methods due to Cohort Study Sites entering the ECHO Study with existing longitudinal data. A primary objective for ECP v3.0 is to standardize data collection while attending to ongoing harmonizability with Cycle 1 data and, simultaneously, recognize when and where improvements to existing Cycle 1 measure collection were necessary. Integrating Cycle 2 data with Cycle 1 data is one of the first primary analytic objectives of the DAC, with support from the Measurement Core. Harmonization efforts will require considering multiple aspects of the data, including constructs measured (e.g., Is the same construct being measured with different instruments?), question-wording (e.g., Do changes in item wording from Cycle 1 to Cycle 2 change the meaning of the item?), response options (e.g., If not the same, can the different response categories be harmonized using algorithmic transformations?), time frame (e.g., Are questions about the past 7 days, past month, past 12 months?), timing of data collection (e.g., Were both measures collected at the same period in the pregnant person or child’s life?), data source (e.g., Was the information collected by medical record abstraction, questionnaire, some other method?), and respondent (e.g., Was data from self-report or proxy? If proxy, is it the same proxy reporter across measurement time points?) [58, 59]. As in Cycle 1, analytical variables will be derived and included with the source variables. The DAC and Measurement Core will continue to provide guidance documents for using data from non-identical measures (i.e., differences in the measurements used to collect the data) [58, 60].

Three analytic considerations applicable to all analyses will be the handling of (1) pooled data across Cycle 1 and Cycle 2; (2) pooled data across Cohort Study Sites with different participant populations (e.g., community sample versus clinically enriched), sample sizes, and ages; [59] and (3) missing data. First, methods to pool data from across Sites will include individual participant data (IPD) meta-analysis with cohort fixed effects to control for any unobserved differences between Cohort Study Sites; [61] accounting for correlation of participants by Site by using generalized estimating equations or Cohort Study Site random intercept in a random effects or multilevel model; and coordinated analysis, which applies similar statistical models to each Cohort Study Site’s raw data and uses meta-analytic techniques to produce overall effect sizes [62, 63]. Such strategies have benefits and drawbacks that will be addressed in individual analysis proposals. For example, to use approaches that pool individual-level data, data must be harmonized across Cohort Study Sites, and pooling greatly enhances sample size [64, 65]. The standardization found in ECP v3.0 facilitates such pooling methods. Alternatively, individual participant data meta-analysis (IPD-MA) analyses can use raw Cohort Study Site data where the same constructs were assessed using different instruments and can investigate heterogeneity across Cohort Study Sites and data collection methods; however, IPD-MA requires separate analysis by cohort, thus relying on original cohort sample sizes versus the combined overall sample size [66, 67]. This latter approach may be most appropriate when combining data from Cycle 2 with data from Cycle 1 when Cohort Study Sites collected various measures at varying child ages and with varying frequencies across prenatal through adolescence to capture the same element. It is of less concern when restricting analyses to Cycle 2 data.

Second, to address missing data, preliminary quality assurance analyses will be performed to identify any systematic patterns of missingness overall and by Cohort Study Site. For data missingness at random, methods such as multiple imputation by chained equations (MICE) [68] within Cohort Study Site or potentially across Study Sites can be employed. Additionally, high levels of Cohort Study Site missing data can be imputed within groups of Sites with similar designs and selection criteria using qualitative combinations of Cohort Study Sites (e.g., combining all participants from Cohort Study Sites recruiting based on NICU admission) or using the RELATE method [69] explicitly developed for this purpose of identifying Sites of similar covariate distributions to improve imputation precision.

Given the collection of individual-level residential address history and annual address updates, the ECHO Cohort will also be able to continue to conduct analyses using geocoded address data. Building on Cycle 1 efforts to integrate area-level data databases (e.g., Social Vulnerability Index [70], Child Opportunities Index [71]) into the ECHO analysis workbench, investigators in Cycle 2 will also be able to investigate neighborhood-level exposures (e.g., light pollution, greenspace, structural racism, food deserts) via geocoded residential address and history data. Integration of additional external databases will continue in Cycle 2.

The ECHO DAC will lead many of the consortium-wide statistical analyses, with support from the Measurement Core and Cohort Study Site investigators. The Lab Core will conduct biospecimen assay analyses, the results of which will then be integrated by the DAC into the ECHO Data Platform for consortium use. Informed by investigator interests and stakeholder priorities, the Lab Core is developing a preliminary list of standard assays, with additional assays conducted to meet specific analysis proposal needs.

### Ethics

The WCG IRB is the properly constituted single IRB (sIRB) of record for ECHO, formally designated with reviewing and monitoring research involving human subjects and is accountable for compliance with regulatory requirements for the ECHO Study. The sIRB reviewed the protocol and all informed consent/assent forms, HIPAA Authorization forms, recruitment materials, and other relevant information prior to the initiation of any protocol-related procedures or activities. The sIRB also will review any amendments to the protocol prior to their implementation. Written informed consent is obtained from all adult participants 18 years and older. For participants <18 years old, written informed parent/guardian permission is obtained along with child assent for ages 6–17 years as appropriate, with some flexibility in the lower age bound of assent depending on local Cohort Study Site regulations. Participation in all data collection is voluntary, and participants can opt not to complete specific measures, including biospecimen collection. Additionally, Cohort Study Site staff are required to complete the ECHO Consent/Assent Limitations form when participants do not want certain information shared for research purposes, such as address information that could identify where they live or using biospecimens for genetics research. Cohort Study Site staff will also track and report on study withdrawal via the ECHO Study Withdrawal form, which details the date, circumstance, and reason for participant withdrawal. While the risk for an adverse event resulting from ECP v3.0 procedures is low, if such an event occurs, Cohort Study Sites must report the event within 24 hours to the ECHO CC and within five business days to the ECHO sIRB. All data related to consent/assent limitations, withdrawal, and adverse events will be reviewed on an ongoing basis to identify any potential systematic patterns that may be ameliorated by amendments to the study design that protect participant safety and do not compromise scientific rigor.

## Discussion

The ECHO Program is poised to be a national research resource for investigating important public health topics meaningful to children and families, clinicians, researchers, and policymakers. With its vast size, age span, and geographic, economic, racial, and ethnic diversity [43], ECHO is the largest longitudinal study of child health in the US.

Because of its size and emphasis on minimizing burden for participants and staff, the ECHO Program is also necessarily limited in that it cannot assess all types of environmental exposures and child health outcomes, nor can it conduct deep phenotyping on every outcome for every participant. The uniqueness of ECHO lies in the Program’s ability to be more than the sum of its parts, assessing a broad and integrated spectrum of exposure and relevant outcomes, which will move the science beyond what any individual pediatric cohort study could achieve.

Moreover, select de-identified data from the ECHO Program are available through NICHD’s Data and Specimen Hub (DASH) [72], thus expanding the possible scientific inquiries the broader research field. Information on study data not available on DASH, such as some Indigenous population datasets, can be found on the ECHO study DASH webpage. Study results developed by ECHO investigators follow the International Committee of Medical Journal Editors (ICMJE) guidelines for authorship, and publications will be disseminated via scientific publications and national conference presentations, public webinars, and the public-facing ECHO Program website, ECHOChildren.org, which includes research summaries in English and Spanish of all ECHO publications. Through its publicly available data and broad dissemination efforts, the ECHO Program aims to inform future research, policies, and practices to improve health for all children.

### Planned protocol updates

A unique component of ECHO Cycle 2 will be the integration of preconception data collection on individuals who may become pregnant again during the study and, when possible, on their current partners. Individuals with the potential for becoming pregnant will be drawn from the prenatal Cohort Study Sites following the first pregnancy, and when available, parenting partners will be invited to participate as well. Development of the preconception section of the protocol is underway, with expected implementation in fall 2024.

Furthermore, specialized data elements, measures, and biospecimens for each exposure and outcome area will be added to the protocol to capture in-depth assessments in subsets of ECHO participants. Specifically, each Cohort Study Site will select at least one child health outcome area—and for prenatal Cohort Study Sites, an exposure area—in which to specialize and collect all measures and biospecimens in their respective specialized areas. The protocol version incorporating specialized data elements, measures, and biospecimens will be finalized in 2024 for implementation in early 2025.

Additionally, the COVID-19 pandemic elucidated the need for a nimble approach to research, and the ECHO Investigators are developing processes based on lessons learned during the pandemic in Cycle 1 to ensure rapid response to important public health events that may arise. ECHO Protocol v3.0 reserves “ad hoc” assessment time in every study site to enable assessment of emergency events. Investigators are also working to identify and devise generic disaster response instruments that can be implemented in real-time to assess participants’ experiences of emergencies and the impact of such events on their physical, mental, and social health. Additionally, work is underway to prioritize the development and implementation of methods for assessing the impact of specific public health emergencies that may occur during Cycle 2 (e.g., wildfires, hurricanes, school shootings, influenza pandemic).

The ECHO investigators further recognize the importance of responding to scientific and technological advancements that may occur during Cycle 2. Annual protocol review will occur to accommodate these advancements and ensure ECHO remains on the leading edge of child health research. Investigators will have opportunities to provide feedback during the annual review, and solicitations for input from the public will be made through NIH Requests for Information. Additionally, ECHO’s External Scientific Board (ESB) acts in an oversight capacity to help ensure the long-term success of the ECHO Program. Comprised of transdisciplinary experts from such fields as maternal and fetal medicine, pediatrics, early childhood development, epidemiology, community-based participatory research, human subjects research, bioethics, and data science, the ECHO ESB holds biannual meetings to review the overall progress of the ECHO Program and advise on strategies to overcome any programmatic challenges. As part of this work, the ESB provides input on protocol development to ensure high-impact child health research that responds to diverse stakeholder interests.

In summary, the ECHO Program is poised to make significant contributions to solution-oriented pediatric health research, policies, and practices. Both the size and scope of ECHO with enable richly characterized phenotyping of priority child health outcomes and the biological, social, and environmental exposures that impact them.

## Supporting information

S1 Table References for ECHO Cohort Data and biospecimen Protocol v3.0 measures. (DOCX)

## References

[1] Shonkoff JP, Boyce WT, McEwen BS. Neuroscience, molecular biology, and the childhood roots of health disparities: building a new framework for health promotion and disease prevention. *Jama*. Jun 3 2009;301(21):2252–9. doi: 10.1001/jama.2009.754 19491187

[2] Marques AH, O’Connor TG, Roth C, Susser E, Bjørke-Monsen AL. The influence of maternal prenatal and early childhood nutrition and maternal prenatal stress on offspring immune system development and neurodevelopmental disorders. *Front Neurosci*. 2013;7:120. doi: 10.3389/fnins.2013.00120 23914151 PMC3728489

[3] McEwen BS. Early life influences on life-long patterns of behavior and health. *Ment Retard Dev Disabil Res Rev*. 2003;9(3):149–54. doi: 10.1002/mrdd.10074 12953293

[4] Rice D, Barone S Jr. Critical periods of vulnerability for the developing nervous system: evidence from humans and animal models. *Environ Health Perspect*. Jun 2000;108 Suppl 3(Suppl 3):511–33. doi: 10.1289/ehp.00108s3511 10852851 PMC1637807

[5] Miller GW, Jones DP. The nature of nurture: refining the definition of the exposome. *Toxicological sciences*. 2014;137(1):1–2. doi: 10.1093/toxsci/kft251 24213143 PMC3871934

[6] Borders AE, Grobman WA, Amsden LB, Holl JL. Chronic stress and low birth weight neonates in a low-income population of women. *Obstet Gynecol*. Feb 2007;109(2 Pt 1):331–8. doi: 10.1097/01.AOG.0000250535.97920.b5 17267833

[7] Harville EW, Mishra GD, Yeung E, et al. The preconception period analysis of risks and exposures influencing health and development (PrePARED) consortium. *Paediatric and perinatal epidemiology*. 2019;33(6):490–502. doi: 10.1111/ppe.12592 31659792 PMC6901022

[8] Grandjean P, Landrigan PJ. Neurobehavioural effects of developmental toxicity. *Lancet Neurol*. Mar 2014;13(3):330–8. doi: 10.1016/S1474-4422(13)70278-3 24556010 PMC4418502

[9] Rauh VA, Margolis AE. Research Review: Environmental exposures, neurodevelopment, and child mental health—new paradigms for the study of brain and behavioral effects. *J Child Psychol Psychiatry*. Jul 2016;57(7):775–93. doi: 10.1111/jcpp.12537 26987761 PMC4914412

[10] Boucher O, Muckle G, Jacobson JL, et al. Domain-specific effects of prenatal exposure to PCBs, mercury, and lead on infant cognition: results from the Environmental Contaminants and Child Development Study in Nunavik. *Environ Health Perspect*. Mar 2014;122(3):310–6. doi: 10.1289/ehp.1206323 24441767 PMC3948023

[11] Clark CA, Espy KA, Wakschlag L. Developmental pathways from prenatal tobacco and stress exposure to behavioral disinhibition. *Neurotoxicol Teratol*. Jan-Feb 2016;53:64–74. doi: 10.1016/j.ntt.2015.11.009 26628107 PMC4889442

[12] Williams DR, Sternthal M, Wright RJ. Social determinants: taking the social context of asthma seriously. *Pediatrics*. Mar 2009;123 Suppl 3(Suppl 3):S174–84. doi: 10.1542/peds.2008-2233H 19221161 PMC3489274

[13] Halfon N, Wise PH, Forrest CB. The changing nature of children’s health development: new challenges require major policy solutions. *Health Aff (Millwood)*. Dec 2014;33(12):2116–24. doi: 10.1377/hlthaff.2014.0944 25489029

[14] Wiebe SA, Clark CA, De Jong DM, Chevalier N, Espy KA, Wakschlag L. Prenatal tobacco exposure and self-regulation in early childhood: Implications for developmental psychopathology. *Dev Psychopathol*. May 2015;27(2):397–409. doi: 10.1017/S095457941500005X 25997761 PMC10112534

[15] Cowell WJ, Brunst KJ, Malin AJ, et al. Prenatal exposure to PM 2. 5 and cardiac vagal tone during infancy: Findings from a multiethnic birth cohort. *Environmental health perspectives*. 2019;127(10):107007. doi: 10.1289/EHP4434 31663780 PMC6867319

[16] Cowell WJ, Colicino E, Tanner E, et al. Prenatal toxic metal mixture exposure and newborn telomere length: modification by maternal antioxidant intake. *Environmental Research*. 2020;190(110009) doi: 10.1016/j.envres.2020.110009 32777275 PMC7530067

[17] Howe CG, Farzan SF, Garcia E, et al. Arsenic and birth outcomes in a predominately lower income Hispanic pregnancy cohort in Los Angeles. *Environmental Research*. 2020;184:109294. doi: 10.1016/j.envres.2020.109294 32145549 PMC7103498

[18] Volk HE, Perera F, Braun JM, et al. Prenatal air pollution exposure and neurodevelopment: A review and blueprint for a harmonized approach within ECHO. *Environ Res*. May 2021;196:110320. doi: 10.1016/j.envres.2020.110320 33098817 PMC8060371

[19] Zhang X, Liu SH, Geron M, et al. Prenatal exposure to PM(2.5) and childhood cognition: Accounting for between-site heterogeneity in a pooled analysis of ECHO cohorts in the Northeastern United States. *Environ Res*. Nov 2022;214(Pt 4):114163. doi: 10.1016/j.envres.2022.114163 36030921 PMC9675417

[20] Lee A, Wright RJ. Prenatal stress and childhood asthma risk: taking a broader view. *Eur Respir J*. Feb 2016;47(2):406–9. doi: 10.1183/13993003.01921-2015 26828054

[21] Haggins A, Patrick S, Demonner S, Davis MM. When coverage expands: children’s health insurance program as a natural experiment in use of health care services. *Acad Emerg Med*. Oct 2013;20(10):1026–32. doi: 10.1111/acem.12236 24127706

[22] Wakschlag LS, Henry DB, Blair RJ, Dukic V, Burns J, Pickett KE. Unpacking the association: Individual differences in the relation of prenatal exposure to cigarettes and disruptive behavior phenotypes. *Neurotoxicol Teratol*. Jan-Feb 2011;33(1):145–54. doi: 10.1016/j.ntt.2010.07.002 21256429 PMC3674557

[23] Arendas K, Qiu Q, Gruslin A. Obesity in pregnancy: pre-conceptional to postpartum consequences. *J Obstet Gynaecol Can*. Jun 2008;30(6):477–488. doi: 10.1016/S1701-2163(16)32863-8 18611299

[24] Doherty DA, Magann EF, Francis J, Morrison JC, Newnham JP. Pre-pregnancy body mass index and pregnancy outcomes. *Int J Gynaecol Obstet*. Dec 2006;95(3):242–7. doi: 10.1016/j.ijgo.2006.06.021 17007857

[25] Siega-Riz AM, Vladutiu CJ, Butera NM, et al. Preconception Diet Quality Is Associated with Birth Weight for Gestational Age Among Women in the Hispanic Community Health Study/Study of Latinos. *J Acad Nutr Diet*. Mar 2021;121(3):458–466. doi: 10.1016/j.jand.2020.09.039 33187928 PMC10807347

[26] Yang W, Zeng L, Cheng Y, et al. The effects of periconceptional risk factor exposure and micronutrient supplementation on birth defects in Shaanxi Province in Western China. *PLoS One*. 2012;7(12):e53429. doi: 10.1371/journal.pone.0053429 23300928 PMC3534073

[27] Yu CK, Teoh TG, Robinson S. Obesity in pregnancy. *Bjog*. Oct 2006;113(10):1117–25. doi: 10.1111/j.1471-0528.2006.00991.x 16903839

[28] Zagré NM, Desplats G, Adou P, Mamadoultaibou A, Aguayo VM. Prenatal multiple micronutrient supplementation has greater impact on birthweight than supplementation with iron and folic acid: a cluster-randomized, double-blind, controlled programmatic study in rural Niger. *Food Nutr Bull*. Sep 2007;28(3):317–27. doi: 10.1177/156482650702800308 17974365

[29] Deng Q, Lu C, Ou C, Chen L, Yuan H. Preconceptional, prenatal and postnatal exposure to outdoor and indoor environmental factors on allergic diseases/symptoms in preschool children. *Chemosphere*. Jun 2016;152:459–67. doi: 10.1016/j.chemosphere.2016.03.032 27003368

[30] Gaskins AJ, Mínguez-Alarcón L, Williams PL, et al. Ambient air pollution and risk of pregnancy loss among women undergoing assisted reproduction. *Environ Res*. Dec 2020;191:110201. doi: 10.1016/j.envres.2020.110201 32937174 PMC7658021

[31] Lu C, Deng L, Ou C, Yuan H, Chen X, Deng Q. Preconceptional and perinatal exposure to traffic-related air pollution and eczema in preschool children. *J Dermatol Sci*. Feb 2017;85(2):85–95. doi: 10.1016/j.jdermsci.2016.11.004 27865567

[32] Zhang Q, Sun S, Sui X, et al. Associations between weekly air pollution exposure and congenital heart disease. *Sci Total Environ*. Feb 25 2021;757:143821. doi: 10.1016/j.scitotenv.2020.143821 33248761

[33] Hertz-Picciotto I, Korrick SA, Ladd-Acosta C, et al. Maternal tobacco smoking and offspring autism spectrum disorder or traits in ECHO cohorts. *Autism Res*. Mar 2022;15(3):551–569. doi: 10.1002/aur.2665 35199959 PMC9304219

[34] Lassi ZS, Imam AM, Dean SV, Bhutta ZA. Preconception care: caffeine, smoking, alcohol, drugs and other environmental chemical/radiation exposure. *Reprod Health*. Sep 26 2014;11 Suppl 3(Suppl 3):S6. doi: 10.1186/1742-4755-11-S3-S6 25415846 PMC4196566

[35] Brunst KJ, Tignor N, Just A, et al. Cumulative lifetime maternal stress and epigenome-wide placental DNA methylation in the PRISM cohort. *Epigenetics*. 2018;13(6):665–681. doi: 10.1080/15592294.2018.1497387 30001177 PMC6291301

[36] Leppert B, Junge KM, Röder S, et al. Early maternal perceived stress and children’s BMI: longitudinal impact and influencing factors. *BMC Public Health*. Oct 30 2018;18(1):1211. doi: 10.1186/s12889-018-6110-5 30376822 PMC6208039

[37] Blackwell CK, Wakschlag L, Krogh-Jespersen S, et al. Pragmatic Health Assessment in Early Childhood: The PROMIS^®^ of Developmentally Based Measurement for Pediatric Psychology. *J Pediatr Psychol*. Apr 1 2020;45(3):311–318. doi: 10.1093/jpepsy/jsz094 31774488 PMC7081936

[38] Cella D, Yount S, Rothrock N, et al. The Patient-Reported Outcomes Measurement Information System (PROMIS): progress of an NIH Roadmap cooperative group during its first two years. *Med Care*. May 2007;45(5 Suppl 1):S3–s11. doi: 10.1097/01.mlr.0000258615.42478.55 17443116 PMC2829758

[39] Bevans KB, Riley AW, Forrest CB. Development of the healthy pathways child-report scales. *Qual Life Res*. Oct 2010;19(8):1195–214. doi: 10.1007/s11136-010-9687-4 20563886 PMC2940033

[40] Gershon RC, Wagster MV, Hendrie HC, Fox NA, Cook KF, Nowinski CJ. NIH toolbox for assessment of neurological and behavioral function. *Neurology*. Mar 12 2013;80(11 Suppl 3):S2–6. doi: 10.1212/WNL.0b013e3182872e5f 23479538 PMC3662335

[41] Gillman MW, Blaisdell CJ. Environmental influences on Child Health Outcomes, a Research Program of the National Institutes of Health. *Curr Opin Pediatr*. Apr 2018;30(2):260–262. doi: 10.1097/MOP.0000000000000600 29356702 PMC6020137

[42] Blaisdell CJ, Park C, Hanspal M, et al. The NIH ECHO Program: investigating how early environmental influences affect child health. *Pediatr Res*. Nov 2022;92(5):1215–1216. doi: 10.1038/s41390-021-01574-8 34131291 PMC8204611

[43] Knapp EA, Kress AM, Parker CB, et al. The Environmental Influences on Child Health Outcomes (ECHO)-Wide Cohort. *Am J Epidemiol*. Aug 4 2023;192(8):1249–1263. doi: 10.1093/aje/kwad071 36963379 PMC10403303

[44] National Institutes of Health. Clinical Sites for the ECHO IDeA States Pediatric Clinical Trials Network. US Department of Health & Human Services, National Institutes of Health. Accessed September 15, 2024, https://www.nih.gov/echo/clinical-sites-echo-idea-states-pediatric-clinical-trials-network

[45] National Institutes of Health. Environmental influences on Child Health Outcomes (ECHO) Program. US Department of Health & Human Services, National Institutes of Health. Accessed September 15, 2024, https://www.nih.gov/research-training/environmental-influences-child-health-outcomes-echo-program

[46] Tierney AL, Nelson CA III. Brain Development and the Role of Experience in the Early Years. *Zero to three*. 2009;30(2):9–13. 23894221 PMC3722610

[47] Erikson EH. *Childhood and society*. Norton; 1950.

[48] Piaget J. The Theory of Stages in Cognitive Development. In: Green D, Ford MP, Flamer GB, eds. *Measurement and Piaget* McGraw-Hill; 1971:1–11.

[49] World Health Organization. Adolescent health and development. World Health Organization. Accessed September 17, 2024, https://www.who.int/news-room/questions-and-answers/item/adolescent-health-and-development

[50] Williams K, Thomson D, Seto I, et al. Standard 6: age groups for pediatric trials. *Pediatrics*. 2012;129(Supplement 3):S153–S160. doi: 10.1542/peds.2012-0055I 22661762

[51] US Department of Health and Human Services, Food and Drug Administration. *Pediatric Expertise for Advisory Panels—Guidance for Industry and FDA Staff*. 2003. https://www.fda.gov/regulatory-information/search-fda-guidance-documents/pediatric-expertise-advisory-panels-guidance-industry-and-fda-staff

[52] Hagan JF, Shaw JS, Duncan PM. *Bright futures*: *Guidelines for health supervision of infants*, *children*, *and adolescents*: *Pocket guide*. 2008.

[53] Hardin AP, Hackell JM, Committee on Practice and Ambulatory Medicine, et al. Age limit of pediatrics. *Pediatrics*. 2017;140(3):e20172151. doi: 10.1542/peds.2017-2151 28827380

[54] White E, Hunt JR, Casso D. Exposure measurement in cohort studies: the challenges of prospective data collection. *Epidemiologic reviews*. 1998;20(1):43–56. doi: 10.1093/oxfordjournals.epirev.a017971 9762508

[55] Harris PA, Taylor R, Thielke R, Payne J, Gonzalez N, Conde JG. Research electronic data capture (REDCap)—a metadata-driven methodology and workflow process for providing translational research informatics support. *Journal of biomedical informatics*. 2009;42(2):377–381. doi: 10.1016/j.jbi.2008.08.010 18929686 PMC2700030

[56] Harris PA, Taylor R, Minor BL, et al. The REDCap consortium: building an international community of software platform partners. *Journal of biomedical informatics*. 2019;95:103208. doi: 10.1016/j.jbi.2019.103208 31078660 PMC7254481

[57] Ho E, Ece B, Tuladhar Z, et al. Remote assessment of key pediatric anthropometric outcomes. *Pediatrics*. under review; 10.1542/peds.2024-067663 PMC12917953 40049218

[58] Fortier I, Wey TW, Bergeron J, et al. Life course of retrospective harmonization initiatives: key elements to consider. *Journal of developmental origins of health and disease*. 2023;14(2):190–198. doi: 10.1017/S2040174422000460 35957574

[59] Lesko CR, Jacobson LP, Althoff KN, et al. Collaborative, pooled and harmonized study designs for epidemiologic research: challenges and opportunities. *Int J Epidemiol*. Apr 1 2018;47(2):654–668. doi: 10.1093/ije/dyx283 29438495 PMC5913631

[60] Fortier I, Raina P, Van den Heuvel ER, et al. Maelstrom Research guidelines for rigorous retrospective data harmonization. *International journal of epidemiology*. 2017;46(1):103–105. doi: 10.1093/ije/dyw075 27272186 PMC5407152

[61] Wooldridge JM. *Econometric Analysis of Cross Section and Panel Data*. MIT Press.; 2002.

[62] Graham EK, Willroth EC, Weston SJ, et al. Coordinated data analysis: Knowledge accumulation in lifespan developmental psychology. *Psychol Aging*. Feb 2022;37(1):125–135. doi: 10.1037/pag0000612 35113619 PMC8814465

[63] Curran PJ, Hussong AM. Integrative data analysis: the simultaneous analysis of multiple data sets. *Psychological methods*. 2009;14(2):81–100. doi: 10.1037/a0015914 19485623 PMC2777640

[64] Thompson A. Thinking big: large-scale collaborative research in observational epidemiology. *European journal of epidemiology*. 2009;24:727–731. doi: 10.1007/s10654-009-9412-1 19967428

[65] Blettner M, Sauerbrei W, Schlehofer B, Scheuchenpflug T, Friedenreich C. Traditional reviews, meta-analyses and pooled analyses in epidemiology. *International journal of epidemiology*. 1999;28(1):1–9. doi: 10.1093/ije/28.1.1 10195657

[66] Debray TP, Moons KG, van Valkenhoef G, et al. Get real in individual participant data (IPD) meta‐analysis: a review of the methodology. *Research synthesis methods*. 2015;6(4):293–309. doi: 10.1002/jrsm.1160 26287812 PMC5042043

[67] Stewart LA, Tierney JF. To IPD or not to IPD? Advantages and disadvantages of systematic reviews using individual patient data. *Evaluation & the health professions*. 2002;25(1):76–97. doi: 10.1177/0163278702025001006 11868447

[68] Jolani S, Debray TP, Koffijberg H, van Buuren S, Moons KG. Imputation of systematically missing predictors in an individual participant data meta‐analysis: a generalized approach using MICE. *Statistics in medicine*. 2015;34(11):1841–1863. doi: 10.1002/sim.6451 25663182

[69] Smirnova E, Zhong Y, Alsaadawi R, et al. Missing data interpolation in integrative multi-cohort analysis with disparate covariate information. *arXiv* 2022:2211.00407.

[70] Flanagan BE, Gregory EW, Hallisey EJ, Heitgerd JL, Lewis B. A social vulnerability index for disaster management. *Journal of Homeland Security and Emergency Management*. 2011;8(1):0000102202154773551792. doi: 10.2202/1547-7355.1792

[71] Noelke C, McArdle N, Baek M, et al. *Child Opportunity Index 2*.*0 Technical Documentation*. 2020. diversitydatakids.org/research- library/research-brief/how-we-built-it

[72] Data and Specimen Hub (DASH). Environmental influences on Child Health Outcomes (ECHO)-wide Cohort. National Insitute of Child Health and Human Development (NICHD). Accessed September 15, 2024, https://dash.nichd.nih.gov/study/424643

