# Supplementary material for: Extending the Environmental influences on Child Health Outcomes (ECHO) Cohort through 2030: Rationale and study protocol
Source: PLoS One. 2024 Dec 26;19(12):e0312677. doi: 10.1371/journal.pone.0312677 (PMC11670998; doi:10.1371/journal.pone.0312677)
Supplement: S1 Table — (DOCX) [file pone.0312677.s001.docx]

**Supporting information**

**S1 Table. References for ECHO Cohort Data and Biospecimen Protocol v3.0 measures**

| **Measure(s)** | **References** | **Publisher / Copyright Holder / Measure Developer / Source Study** |
| --- | --- | --- |
| Ages & Stages Questionnaires, Third Edition (ASQ®-3) | Squires J, Bricker DD. Ages & Stages Questionnaires®, Third Edition (ASQ®-3): A Parent-Completed Child Monitoring System. Baltimore: Paul H. Brookes Publishing Co., Inc; 2009. | Brookes publishing, licensed by the Measurement Core |
| Airways Questionnaire - 1-5 yr olds | Adapted from the International Study of Asthma and Allergies in Childhood (ISAAC). Items with * are from the ISAAC, and items with ** are items that were modified from original ISAAC wording. Asher MI, Keil U, Anderson HR, Beasley R, Crane J, Martinez F, Mitchell EA, Pearce N, Sibbald B, Stewart AW, et al. International Study of Asthma and Allergies in Childhood (ISAAC): rationale and methods. Eur Respir J. 1995 Mar;8(3):483-91. doi: 10.1183/09031936.95.08030483. Beasley R. Worldwide variation in prevalence of symptoms of asthma, allergic rhinoconjunctivitis, and atopic eczema: ISAAC. The Lancet. 1998 Apr 25;351(9111):1225-32. | International Study of Asthma and Allergies in Childhood (ISAAC) |
| Airways Questionnaire - 11-20 yr olds | Adapted from the International Study of Asthma and Allergies in Childhood (ISAAC). Items with * are from the ISAAC, and items with ** are items that were modified from original ISAAC wording. Asher MI, Keil U, Anderson HR, Beasley R, Crane J, Martinez F, Mitchell EA, Pearce N, Sibbald B, Stewart AW, et al. International Study of Asthma and Allergies in Childhood (ISAAC): rationale and methods. Eur Respir J. 1995 Mar;8(3):483-91. doi: 10.1183/09031936.95.08030483. Beasley R. Worldwide variation in prevalence of symptoms of asthma, allergic rhinoconjunctivitis, and atopic eczema: ISAAC. The Lancet. 1998 Apr 25;351(9111):1225-32. | International Study of Asthma and Allergies in Childhood (ISAAC) |
| Airways Questionnaire - 6-10 yr olds | Adapted from the International Study of Asthma and Allergies in Childhood (ISAAC). Items with * are from the ISAAC, and items with ** are items that were modified from original ISAAC wording. Asher MI, Keil U, Anderson HR, Beasley R, Crane J, Martinez F, Mitchell EA, Pearce N, Sibbald B, Stewart AW, et al. International Study of Asthma and Allergies in Childhood (ISAAC): rationale and methods. Eur Respir J. 1995 Mar;8(3):483-91. doi: 10.1183/09031936.95.08030483. Beasley R. Worldwide variation in prevalence of symptoms of asthma, allergic rhinoconjunctivitis, and atopic eczema: ISAAC. The Lancet. 1998 Apr 25;351(9111):1225-32. | International Study of Asthma and Allergies in Childhood (ISAAC) |
| Airways Questionnaire - 6-11 mo olds | Adapted from the International Study of Asthma and Allergies in Childhood (ISAAC). Items with * are from the ISAAC, and items with ** are items that were modified from original ISAAC wording. Asher MI, Keil U, Anderson HR, Beasley R, Crane J, Martinez F, Mitchell EA, Pearce N, Sibbald B, Stewart AW, et al. International Study of Asthma and Allergies in Childhood (ISAAC): rationale and methods. Eur Respir J. 1995 Mar;8(3):483-91. doi: 10.1183/09031936.95.08030483. Beasley R. Worldwide variation in prevalence of symptoms of asthma, allergic rhinoconjunctivitis, and atopic eczema: ISAAC. The Lancet. 1998 Apr 25;351(9111):1225-32. | International Study of Asthma and Allergies in Childhood (ISAAC) |
| Alabama Parenting Questionnaire - 9-Item Child Self-Report | Gross TJ, Fleming CB, Mason WA, Haggerty KP. Alabama Parenting Questionnaire–9: Longitudinal measurement invariance across parents and youth during the transition to high school. Assessment. 2017 Jul;24(5):646-59. Frick PJ, Christian RE, Wootton JM. Age trends in the association between parenting practices and conduct problems. Behavior modification. 1999 Jan;23(1):106-28. Shelton KK, Frick PJ, Wootton J. Assessment of parenting practices in families of elementary school-age children. Journal of clinical child psychology. 1996 Sep 1;25(3):317-29. | Paul Frick https://faculty.lsu.edu/pfricklab/alabamaparenting.php |
| Alabama Parenting Questionnaire - 9-Item Parent-Report | Gross TJ, Fleming CB, Mason WA, Haggerty KP. Alabama Parenting Questionnaire–9: Longitudinal measurement invariance across parents and youth during the transition to high school. Assessment. 2017 Jul;24(5):646-59. Elgar FJ, Waschbusch DA, Dadds MR, Sigvaldason N. Development and validation of a short form of the Alabama Parenting Questionnaire. Journal of Child and Family Studies. 2007 Apr 1;16(2):243-59. Shelton KK, Frick PJ, Wootton J. Assessment of parenting practices in families of elementary school-age children. Journal of clinical child psychology. 1996 Sep 1;25(3):317-29. | Used with permission of the author of the copyright protected items, Paul Frick. https://faculty.lsu.edu/pfricklab/alabamaparenting.php |
| Alabama Parenting Questionnaire – Preschool Short Form | Clerkin SM, Halperin JM, Marks DJ, Policaro KL. Psychometric properties of the Alabama parenting questionnaire–preschool revision. Journal of Clinical Child and Adolescent Psychology. 2007 Mar 1;36(1):19-28. de la Osa N, Granero R, Penelo E, Domènech JM, Ezpeleta L. Psychometric properties of the Alabama parenting questionnaire-preschool revision (APQ-Pr) in 3 year-old Spanish preschoolers. Journal of Child and Family Studies. 2014 Jul;23:776-84. | Used with permission of the author of the copyright protected items, Paul Frick. https://faculty.lsu.edu/pfricklab/alabamaparenting.php |
| Alabama Parenting Questionnaire – Toddler Short Form | Modified from: Clerkin, S. M., Halperin, J. M., Marks, D. J., & Policaro, K. L. (2007). Psychometric properties of the Alabama parenting questionnaire–preschool revision. Journal of Clinical Child and Adolescent Psychology, 36(1), 19-28; de la Osa, N., Granero, R., Penelo, E., Domènech, J. M., & Ezpeleta, L. (2014). Psychometric properties of the Alabama parenting questionnaire-preschool revision (APQ-Pr) in 3 year-old Spanish preschoolers. Journal of Child and Family Studies, 23, 776-784. | Used modified with permission of the author of the copyright protected items, Paul Frick. https://faculty.lsu.edu/pfricklab/alabamaparenting.php |
| Child Behavior Checklist for Ages 1½-5 (CBCL/1½-5) | Achenbach TM, Rescorla LA. Manual for the ASEBA preschool forms & profiles. Burlington: University of Vermont, Research Center for Children, Youth & Families; 2000. | ASEBA, licensed by the Measurement Core |
| Child Behavior Checklist for Ages 6-18 (CBCL/6-18) | Achenbach TM. Manual for ASEBA school-age forms & profiles. Burlington: University of Vermont, Research Center for Children, Youth & Families 2001. | ASEBA, licensed by the Measurement Core |
| Couples Satisfaction Index 4-item | Funk JL, Rogge RD. Testing the ruler with item response theory: increasing precision of measurement for relationship satisfaction with the Couples Satisfaction Index. Journal of family psychology. 2007 Dec;21(4):572. | Funk & Rogge, 2007 |
| Crisis in the Family Systems - Revised (CRISYS-R) Short Form | Sherlock P, Shalowitz MU, Berry C, Cella D, Blackwell CK, Cowell W, Rodriguez KM, Wright RJ, program collaborators for Environmental Influences on Child Health Outcomes. A short form of the Crisis in Family Systems (CRISYS) in a racially diverse sample of pregnant women. Current Psychology. 2023 Apr 1:1-9.  Shalowitz MU, Berry CA, Rasinski KA, Dannhausen-Brun CA. A new measure of contemporary life stress: development, validation, and reliability of the CRISYS. Health services research. 1998 Dec;33(5 Pt 1):1381.  Berry C, Quinn K, Shalowitz M, Wolf R. Validation of the crisis in family systems–revised, a contemporary measure of life stressors. Psychological Reports. 2001 Jun;88(3):713-24. | Berry et al., 2001; Shalowitz et al., 1998 |
| Dietary Screener Questionnaire - Parent Report | National Cancer Institute. Dietary Screener Questionnaire (DSQ) in the NHANES 2009–10: DSQ.  See NCI website for full list of references: https://epi.grants.cancer.gov/nhanes/dietscreen/evaluation.html#pub | National Cancer Institute http://epi.grants.cancer.gov/nhanes/dietscreen/questionnaires.html |
| Dietary Screener Questionnaire - Self Report | National Cancer Institute. Dietary Screener Questionnaire (DSQ) in the NHANES 2009–10: DSQ.  See NCI website for full list of references: https://epi.grants.cancer.gov/nhanes/dietscreen/evaluation.html#pub | National Cancer Institute http://epi.grants.cancer.gov/nhanes/dietscreen/questionnaires.html |
| Healthy Pathways Bullying Scales | Bevans KB, Riley AW, Forrest CB. Development of the healthy pathways child-report scales. Quality of Life Research. 2010 Oct 1;19(8):1195-1214. | Bevans et al. 2010 |
| Household Chemical Exposure - Current | Adapted from the Early Life Exposures Assessment Tool (ELEAT)© | Rebecca J. Schmidt, Cheryl Walker, and Debbie Bennett. Copyright © The Regents of the University of California, Davis campus 2012-23. All Rights Reserved. Used with permission. |
| Household Chemical Exposure - Pre-pregnancy & Pregnancy | Adapted from the Early Life Exposures Assessment Tool (ELEAT)© | Rebecca J. Schmidt, Cheryl Walker, and Debbie Bennett. Copyright © The Regents of the University of California, Davis campus 2012-23. All Rights Reserved. Used with permission. |
| Household Exposure to Secondhand Smoke - Current | Adapted from the Early Life Exposures Assessment Tool (ELEAT)© | Rebecca J. Schmidt, Cheryl Walker, and Debbie Bennett. Copyright © The Regents of the University of California, Davis campus 2012-23. All Rights Reserved. Used with permission. |
| Household Exposure to Secondhand Smoke - Current - 18-20 yr olds | Adapted from the Early Life Exposures Assessment Tool (ELEAT)© | Rebecca J. Schmidt, Cheryl Walker, and Debbie Bennett. Copyright © The Regents of the University of California, Davis campus 2012-23. All Rights Reserved. Used with permission. |
| Household Exposure to Secondhand Smoke - Pre-pregnancy & Pregnancy | Adapted from the Early Life Exposures Assessment Tool (ELEAT)© | Rebecca J. Schmidt, Cheryl Walker, and Debbie Bennett. Copyright © The Regents of the University of California, Davis campus 2012-23. All Rights Reserved. Used with permission. |
| Income, Assistance, and Financial Strain  - 18-20 yr olds | Section B, items 19-23 were modified U.S. Household Food Security Survey Module: Six-Item Short Form | US Department of Agriculture https://www.ers.usda.gov/media/8282/short2012.pdf |
| Income, Assistance, and Financial Strain - Childhood | Items 13-24 were modified U.S. Household Food Security Survey Module: Six-Item Short Form (items 13-17) and Child Food Security Module (items 18-24) | US Department of Agriculture https://www.ers.usda.gov/media/8282/short2012.pdf https://www.ers.usda.gov/media/8271/hh2012.pdf |
| Income, Assistance, and Financial Strain - Pregnancy | Items 14-18 were modified U.S. Household Food Security Survey Module: Six-Item Short Form | US Department of Agriculture  https://www.ers.usda.gov/media/8282/short2012.pdf |
| Infant Feeding Practices | Adapted from the Infant Feeding Practices Study II postnatal questionnaires | Center for Disease Control and Prevention (CDC) https://www.cdc.gov/breastfeeding/data/ifps/questionnaires.htm |
| Language and Acculturation - 18-20 yr olds | Acculturation questions adapted from the PhenX Toolkit #180101, Ver 24.0 and PhenX Toolkit #270201, Ver.44.1.   #180101: Alegria, M., Vila, D., Woo, M., Canino, G., Takeuchi, D., Vera, M., Febo, V., Guarnaccia, P., Aguilar-Gaxiola, S., & Shrout, P. (2004). Cultural relevance and equivalence in the NLAAS instrument: Integrating etic and emic in the development of cross-cultural measures for a psychiatric epidemiology and services study of Latinos. International Journal of Methods in Psychiatric Research, 13(4), 270-288. Guarnaccia, P. J., Pincay, I. M., Alegria, M., Shrout, P., Lewis-Fernandez, R., & Canino, G. (2007). Assessing diversity among Latinos: Results from the NLAAS. Hispanic Journal of Behavioral Sciences, 29(4), 510-534. Kagawa-Singer, M., Dressler, W. W., George, S. M., & Elwood, W. N. (2015). The cultural framework for health: An integrative approach for research and program design and evaluation. Bethesda, MD: National Institutes of Health, Office of Behavioral and Social Sciences Research.  #270201: See PhenX for full list of references. https://www.phenxtoolkit.org/protocols/view/270201 U.S. Census Bureau. (2020). American Community Survey (ACS), 2020. Washington, DC: Author. | PhenX Toolkit: https://www.phenxtoolkit.org/  National Institutes of Health, National Institute of Mental Health. (2002). National Latino and Asian American Study (NLAAS), 2002. Question numbers LP5d, LP5e, LP5f, LP7a, LP7b, and LP7c are represented in this protocol as 1, 2, 3, 5, 6, and 7, respectively.  Regents of the University of California. (2019). CHIS 2018 Adult Questionnaire, question number “QA18_G8” is represented in this protocol as question 1. Retrieved from http://healthpolicy.ucla.edu/chis/design/Pages/questionnairesEnglish.aspx |
| Language and Acculturation - Childhood | Acculturation questions adapted from the PhenX Toolkit #180101, Ver 24.0 and PhenX Toolkit #270201, Ver.44.1. #180101: Alegria, M., Vila, D., Woo, M., Canino, G., Takeuchi, D., Vera, M., Febo, V., Guarnaccia, P., Aguilar-Gaxiola, S., & Shrout, P. (2004). Cultural relevance and equivalence in the NLAAS instrument: Integrating etic and emic in the development of cross-cultural measures for a psychiatric epidemiology and services study of Latinos. International Journal of Methods in Psychiatric Research, 13(4), 270-288. Guarnaccia, P. J., Pincay, I. M., Alegria, M., Shrout, P., Lewis-Fernandez, R., & Canino, G. (2007). Assessing diversity among Latinos: Results from the NLAAS. Hispanic Journal of Behavioral Sciences, 29(4), 510-534. Kagawa-Singer, M., Dressler, W. W., George, S. M., & Elwood, W. N. (2015). The cultural framework for health: An integrative approach for research and program design and evaluation. Bethesda, MD: National Institutes of Health, Office of Behavioral and Social Sciences Research.  #270201: See PhenX for full list of references. https://www.phenxtoolkit.org/protocols/view/270201 U.S. Census Bureau. (2020). American Community Survey (ACS), 2020. Washington, DC: Author. | PhenX Toolkit: https://www.phenxtoolkit.org/  National Institutes of Health, National Institute of Mental Health. (2002). National Latino and Asian American Study (NLAAS), 2002. Question numbers LP5d, LP5e, LP5f, LP7a, LP7b, and LP7c are represented in this protocol as 1, 2, 3, 5, 6, and 7, respectively.  Regents of the University of California. (2019). CHIS 2018 Adult Questionnaire, question number “QA18_G8” is represented in this protocol as question 1. Retrieved from http://healthpolicy.ucla.edu/chis/design/Pages/questionnairesEnglish.aspx |
| Language and Acculturation - Pregnancy | Acculturation questions adapted from the PhenX Toolkit #180101, Ver 24.0 and PhenX Toolkit #270201, Ver.44.1. https://www.phenxtoolkit.org/  #180101: Alegria, M., Vila, D., Woo, M., Canino, G., Takeuchi, D., Vera, M., Febo, V., Guarnaccia, P., Aguilar-Gaxiola, S., & Shrout, P. (2004). Cultural relevance and equivalence in the NLAAS instrument: Integrating etic and emic in the development of cross-cultural measures for a psychiatric epidemiology and services study of Latinos. International Journal of Methods in Psychiatric Research, 13(4), 270-288. Guarnaccia, P. J., Pincay, I. M., Alegria, M., Shrout, P., Lewis-Fernandez, R., & Canino, G. (2007). Assessing diversity among Latinos: Results from the NLAAS. Hispanic Journal of Behavioral Sciences, 29(4), 510-534. Kagawa-Singer, M., Dressler, W. W., George, S. M., & Elwood, W. N. (2015). The cultural framework for health: An integrative approach for research and program design and evaluation. Bethesda, MD: National Institutes of Health, Office of Behavioral and Social Sciences Research.  #270201: See PhenX for full list of references. https://www.phenxtoolkit.org/protocols/view/270201 U.S. Census Bureau. (2020). American Community Survey (ACS), 2020. Washington, DC: Author. | PhenX Toolkit: https://www.phenxtoolkit.org/  National Institutes of Health, National Institute of Mental Health. (2002). National Latino and Asian American Study (NLAAS), 2002. Question numbers LP5d, LP5e, LP5f, LP7a, LP7b, and LP7c are represented in this protocol as 1, 2, 3, 5, 6, and 7, respectively.  Regents of the University of California. (2019). CHIS 2018 Adult Questionnaire, question number “QA18_G8” is represented in this protocol as question 1. Retrieved from http://healthpolicy.ucla.edu/chis/design/Pages/questionnairesEnglish.aspx |
| Life Stressor Checklist - Revised (LSC-R) | Modified from: Wolfe J, Kimerling R, Brown PJ, Chrestman KR, Levin K. Life Stressor Checklist--Revised. Journal of Community Psychology. 1996. | Wolfe et al., 1997  Available from https://www.ptsd.va.gov/ |
| Lifestyle - 3rd Trimester | Adapted from the Early Life Exposures Assessment Tool (ELEAT)© | Rebecca J. Schmidt, Cheryl Walker, and Debbie Bennett. Copyright © The Regents of the University of California, Davis campus 2012-23. All Rights Reserved. Used with permission. |
| Lifestyle - Childhood | Adapted from the Early Life Exposures Assessment Tool (ELEAT)© | Rebecca J. Schmidt, Cheryl Walker, and Debbie Bennett. Copyright © The Regents of the University of California, Davis campus 2012-23. All Rights Reserved. Used with permission. |
| Lifestyle - Early Pregnancy | Adapted from the Early Life Exposures Assessment Tool (ELEAT)© | Rebecca J. Schmidt, Cheryl Walker, and Debbie Bennett. Copyright © The Regents of the University of California, Davis campus 2012-23. All Rights Reserved. Used with permission. |
| Lifestyle - Partner | Adapted from the Early Life Exposures Assessment Tool (ELEAT)© | Rebecca J. Schmidt, Cheryl Walker, and Debbie Bennett. Copyright © The Regents of the University of California, Davis campus 2012-23. All Rights Reserved. Used with permission. |
| Lifestyle - Substance Use Recall | Adapted from the Early Life Exposures Assessment Tool (ELEAT)© | Rebecca J. Schmidt, Cheryl Walker, and Debbie Bennett. Copyright © The Regents of the University of California, Davis campus 2012-23. All Rights Reserved. Used with permission. |
| Maternal Supplements Short Form - Pregnancy | Adapted from the Early Life Exposures Assessment Tool (ELEAT)© | Rebecca J. Schmidt, Cheryl Walker, and Debbie Bennett. Copyright © The Regents of the University of California, Davis campus 2012-23. All Rights Reserved. Used with permission. |
| Maternal Supplements Short Form – Pregnancy Partner Supplements | Adapted from the Early Life Exposures Assessment Tool (ELEAT)© | Rebecca J. Schmidt, Cheryl Walker, and Debbie Bennett. Copyright © The Regents of the University of California, Davis campus 2012-23. All Rights Reserved. Used with permission. |
| Modified Checklist for Autism in Toddlers Revised (M-CHAT-R) | Modified from the M-CHAT-R ©2009 Diana Robins, Deborah Fein, & Marianne Barton.  Robins DL, Casagrande K, Barton M, Chen CM, Dumont-Mathieu T, Fein D. Validation of the modified checklist for autism in toddlers, revised with follow-up (M-CHAT-R/F). Pediatrics. 2014 Jan 1;133(1):37-45. | Diane L. Robins |
| Neighborhood Collective Efficacy - Pregnant Person/Caregiver | PhenX Toolkit version August 1, 2018, Ver 24.0. Neighborhood Collective Efficacy (#210801), available from www.phenxtoolkit.org.    Exemplar use in large scale studies (see PhenX Toolkit for full list of references: https://www.phenxtoolkit.org/protocols/view/210801) ABCD: Gonzalez R, Thompson EL, Sanchez M, Morris A, Gonzalez MR, Ewing SW, Mason MJ, Arroyo J, Howlett K, Tapert SF, Zucker RA. An update on the assessment of culture and environment in the ABCD Study®: Emerging literature and protocol updates over three measurement waves. Developmental cognitive neuroscience. 2021 Dec 1;52:101021. Fagile Families: Reichman N.E., Teitler J.O., Garfinkel I., McLanahan S.S. Fragile families: sample and design. Children Youth Serv. Rev. 2001;23(4–5):303–326. doi: 10.1016/S0190-7409(01)00141-4.  Reliability and Validity: Sampson R.J., Raudenbush S.W., Earls F. Neighborhoods and violent crime: a multilevel study of collective efficacy. Science. 1997;277(5328):918–924. doi: 10.1126/science.277.5328.918. | National Archive of Criminal Justice Data (NACJD), Project on Human Development in Chicago Neighborhoods (PHDCN). Community Survey 1994-1995. Questions 11b, 11e, 11f, 11k, 11m (Questions 1-5) and 12a-12c, 12e, 12f (Questions 6-10). |
| NIH Mobile Toolbox v3 | Gershon RC, Sliwinski MJ, Mangravite L, et al. The Mobile Toolbox for monitoring cognitive function. Lancet Neurol. 2022;21(7):589-590. doi:10.1016/S1474-4422(22)00225-3 | © 2023 Toolbox Assessments, Inc. All rights reserved. |
| NIH Toolbox 2-minute walk test V3 | Reuben DB, Magasi S, McCreath HE, et al. Motor assessment using the NIH Toolbox. Neurology. 2013;80(11 Suppl 3):S65-S75. doi:10.1212/WNL.0b013e3182872e01 | © 2023 Toolbox Assessments, Inc. All rights reserved. |
| NIH Toolbox V3 Early Cognition Battery | Gershon RC, Wagster MV, Hendrie HC, Fox NA, Cook KF, Nowinski CJ. NIH toolbox for assessment of neurological and behavioral function. Neurology. 2013;80(11 Suppl 3):S2-S6. doi:10.1212/WNL.0b013e3182872e5f | © 2023 Toolbox Assessments, Inc. All rights reserved. |
| NIH Toolbox Cognition Battery v3 | Gershon RC, Waister MV, Hendrie HC, Fox NA, Cook KF, Nowinski CJ. NIH toolbox for assessment of neurological and behavioral function. Neurology. 2013;80(11 Suppl 3):S2-S6. doi:10.1212/WNL.0b013e3182872e5f | © 2023 Toolbox Assessments, Inc. All rights reserved. |
| NIH Toolbox v2.0 - Emotional Support - 18-20 yr olds/CAT | Measure Name: NIH Toolbox v2.0 - Emotional Support (Ages 18+) - Fixed Form Salsman JM, Butt Z, Pilkonis PA, Cyranowski JM, Zill N, Hendrie HC, Kupst MJ, Kelly MA, Bode RK, Choi SW, Lai JS. Emotion assessment using the NIH Toolbox. Neurology. 2013 Mar 12;80(11 Supplement 3):S76-86. | © 2023 Toolbox Assessments, Inc. All rights reserved. |
| NIH Toolbox v2.0 - Emotional Support - 8-17 yr olds/CAT | Measure Name: NIH Toolbox v2.0 - Emotional Support (Ages 8-17) - Fixed Form Salsman JM, Butt Z, Pilkonis PA, Cyranowski JM, Zill N, Hendrie HC, Kupst MJ, Kelly MA, Bode RK, Choi SW, Lai JS. Emotion assessment using the NIH Toolbox. Neurology. 2013 Mar 12;80(11 Supplement 3):S76-86. | © 2023 Toolbox Assessments, Inc. All rights reserved. |
| NIH Toolbox v2.0 - Perceived Stress Scale 10-Item | Measure Name: NIH Toolbox Fixed Form v2.0 – Perceived Stress (Ages 18+)  *Note this is the same as Cohen’s Perceived Stress Scale 10-item Cohen, S., Kamarck, T., and Mermelstein, R. (1983). A global measure of perceived stress. Journal of Health and Social Behavior, 24, 386-396. Cohen, S., & Williamson, G. (1988). Perceived stress in a probability sample of the United States. In S. Spacapam & S. Oskamp (Eds.), The social psychology of health: Claremont Symposium on applied social psychology. Newbury Park, CA: Sage. | © 2023 Toolbox Assessments, Inc. All rights reserved. |
| NIH Toolbox v2.0 - Perceived Stress Scale 10-Item - 18-20 yr olds | Measure Name: NIH Toolbox Fixed Form v2.0 – Perceived Stress (Ages 18+)  *Note this is the same as Cohen’s Perceived Stress Scale 10-itemCohen, S., Kamarck, T., and Mermelstein, R. (1983). A global measure of perceived stress. Journal of Health and Social Behavior, 24, 386-396. Cohen, S., & Williamson, G. (1988). Perceived stress in a probability sample of the United States. In S. Spacapam & S. Oskamp (Eds.), The social psychology of health: Claremont Symposium on applied social psychology. Newbury Park, CA: Sage. | © 2023 Toolbox Assessments, Inc. All rights reserved. |
| Pregnancy Medical Conditions & Interventions | Adapted from the Early Life Exposures Assessment Tool (ELEAT)© | Rebecca J. Schmidt, Cheryl Walker, and Debbie Bennett. Copyright © The Regents of the University of California, Davis campus 2012-23. All Rights Reserved. Used with permission. |
| PROMIS - Anxiety 8a/CAT | Measure Name: PROMIS v1.0 – Emotional Distress – Anxiety Short Form 8a Pilkonis PA, Choi SW, Reise SP, Stover AM, Riley WT, Cella D, PROMIS Cooperative Group. Item banks for measuring emotional distress from the Patient-Reported Outcomes Measurement Information System (PROMIS®): depression, anxiety, and anger. Assessment. 2011 Sep;18(3):263-83. | © 2010-2023 PROMIS Health Organization (PHO) |
| PROMIS - Depression 8a/CAT | Measure Name: PROMIS v1.0 - Depression Short Form 8a Pilkonis PA, Choi SW, Reise SP, Stover AM, Riley WT, Cella D, PROMIS Cooperative Group. Item banks for measuring emotional distress from the Patient-Reported Outcomes Measurement Information System (PROMIS®): depression, anxiety, and anger. Assessment. 2011 Sep;18(3):263-83. | © 2010-2023 PROMIS Health Organization (PHO) |
| PROMIS - Emotional Support 4a/CAT | Measure Name: PROMIS v2.0 - Emotional Support Short Form 4a  Hahn EA, DeWalt DA, Bode RK, Garcia SF, DeVellis RF, Correia H, Cella D. New English and Spanish social health measures will facilitate evaluating health determinants. Health Psychology. 2014 May;33(5):490. | © 2010-2023 PROMIS Health Organization (PHO) |
| PROMIS - Informational Support 4a/CAT | Measure Name: PROMIS v2.0 - Informational Support Short Form 4a Hahn EA, DeWalt DA, Bode RK, Garcia SF, DeVellis RF, Correia H, Cella D. New English and Spanish social health measures will facilitate evaluating health determinants. Health Psychology. 2014 May;33(5):490. | © 2010-2023 PROMIS Health Organization (PHO) |
| PROMIS - Instrumental Support 4a/CAT | Measure Name: PROMIS v2.0 - Instrumental Support Short Form 4a Hahn EA, DeWalt DA, Bode RK, Garcia SF, DeVellis RF, Correia H, Cella D. New English and Spanish social health measures will facilitate evaluating health determinants. Health Psychology. 2014 May;33(5):490. | © 2010-2023 PROMIS Health Organization (PHO) |
| PROMIS v1.0 - Anxiety 8a - 18-20 yr olds/CAT | Measure Name: PROMIS v1.0 – Emotional Distress – Anxiety Short Form 8a Pilkonis PA, Choi SW, Reise SP, Stover AM, Riley WT, Cella D, PROMIS Cooperative Group. Item banks for measuring emotional distress from the Patient-Reported Outcomes Measurement Information System (PROMIS®): depression, anxiety, and anger. Assessment. 2011 Sep;18(3):263-83. | © 2010-2023 PROMIS Health Organization (PHO) |
| PROMIS v1.0 - Depression 8a - 18-20 yr olds/CAT | Measure Name: PROMIS v1.0 - Depression Short Form 8a Pilkonis PA, Choi SW, Reise SP, Stover AM, Riley WT, Cella D, PROMIS Cooperative Group. Item banks for measuring emotional distress from the Patient-Reported Outcomes Measurement Information System (PROMIS®): depression, anxiety, and anger. Assessment. 2011 Sep;18(3):263-83. | © 2010-2023 PROMIS Health Organization (PHO) |
| PROMIS v1.0 - Engagement - Curiosity 6a - Early Childhood Parent Report | Measure Name: PROMIS® Early Childhood Parent Report Scale v1.0 – Engagement – Curiosity 6a Blackwell CK, Kallen MA, Lai JS, Bevans KB, Wakschlag LS, Cella D. Measuring PROMIS® well-being in early childhood. Journal of pediatric psychology. 2022 Jun;47(5):559-72 | © 2010-2023 PROMIS Health Organization (PHO) |
| PROMIS v1.0 - Family Relationships 4a - Parent Proxy/CAT | Measure Name: PROMIS Parent Proxy v1.0 - Family Relationships Short Form 4a Bevans KB, Riley AW, Landgraf JM, Carle AC, Teneralli RE, Fiese BH, Meltzer LJ, Ettinger AK, Becker BD, Forrest CB. Children’s family experiences: development of the PROMIS® pediatric family relationships measures. Quality of Life Research. 2017 Nov;26:3011-23. | © 2010-2023 PROMIS Health Organization (PHO) |
| PROMIS v1.0 - Family Relationships 4a - Pediatric/CAT | Measure Name: PROMIS Pediatric v1.0 - Family Relationships Short Form 4a Bevans KB, Riley AW, Landgraf JM, Carle AC, Teneralli RE, Fiese BH, Meltzer LJ, Ettinger AK, Becker BD, Forrest CB. Children’s family experiences: development of the PROMIS® pediatric family relationships measures. Quality of Life Research. 2017 Nov;26:3011-23. | © 2010-2023 PROMIS Health Organization (PHO) |
| PROMIS v1.0 - General Life Satisfaction 5a - 18-20 yr olds/CAT | Measure Name: PROMIS v1.0 - General Life Satisfaction Short Form 5a (18-21 yr olds) Salsman JM, Lai JS, Hendrie HC, Butt Z, Zill N, Pilkonis PA, Peterson C, Stoney CM, Brouwers P, Cella D. Assessing psychological well-being: self-report instruments for the NIH Toolbox. Quality of Life Research. 2014 Feb;23:205-15. | © 2010-2023 PROMIS Health Organization (PHO) |
| PROMIS v1.0 - General Life Satisfaction 5a/CAT | Measure Name: PROMIS v1.0 - General Life Satisfaction Short Form 5a  Salsman JM, Lai JS, Hendrie HC, Butt Z, Zill N, Pilkonis PA, Peterson C, Stoney CM, Brouwers P, Cella D. Assessing psychological well-being: self-report instruments for the NIH Toolbox. Quality of Life Research. 2014 Feb;23:205-15. | © 2010-2023 PROMIS Health Organization (PHO) |
| PROMIS v1.0 - Global Health Scale 7+2 - Parent Proxy | Measure Name: PROMIS Parent Proxy Scale v1.0 - Global Health 7 plus 2 Forrest CB, Bevans KB, Pratiwadi R, Moon J, Teneralli RE, Minton JM, Tucker CA. Development of the PROMIS® pediatric global health (PGH-7) measure. Quality of Life Research. 2014 May;23:1221-31. | © 2010-2023 PROMIS Health Organization (PHO) |
| PROMIS v1.0 - Global Health Scale 7+2 - Pediatric | Measure Name: PROMIS Pediatric Scale v1.0 - Global Health 7 plus 2 Forrest CB, Bevans KB, Pratiwadi R, Moon J, Teneralli RE, Minton JM, Tucker CA. Development of the PROMIS® pediatric global health (PGH-7) measure. Quality of Life Research. 2014 May;23:1221-31. | © 2010-2023 PROMIS Health Organization (PHO) |
| PROMIS v1.0 - Global Health Scale 8a - Early Childhood Parent Report | Measure Name: PROMIS Early Childhood Parent Report Scale v1.0 - Global Health Kallen MA, Lai JS, Blackwell CK, Schuchard JR, Forrest CB, Wakschlag LS, Cella D. Measuring Promis® global health in early childhood. Journal of pediatric psychology. 2022 Jun 1;47(5):523-33. | © 2010-2023 PROMIS Health Organization (PHO) |
| PROMIS v1.0 - Life Satisfaction 8b - Parent Proxy/CAT | Measure Name: PROMIS Parent Proxy v1.0 - Life Satisfaction Short Form 8b Forrest CB, Devine J, Bevans KB, Becker BD, Carle AC, Teneralli RE, Moon J, Tucker CA, Ravens-Sieberer U. Development and psychometric evaluation of the PROMIS Pediatric Life Satisfaction item banks, child-report, and parent-proxy editions. Quality of Life Research. 2018 Jan;27:217-34. | © 2010-2023 PROMIS Health Organization (PHO) |
| PROMIS v1.0 - Life Satisfaction 8b - Pediatric/CAT | Measure Name: PROMIS Pediatric v1.0 - Life Satisfaction Short Form 8b Forrest CB, Devine J, Bevans KB, Becker BD, Carle AC, Teneralli RE, Moon J, Tucker CA, Ravens-Sieberer U. Development and psychometric evaluation of the PROMIS Pediatric Life Satisfaction item banks, child-report, and parent-proxy editions. Quality of Life Research. 2018 Jan;27:217-34. | © 2010-2023 PROMIS Health Organization (PHO) |
| PROMIS v1.0 - Meaning and Purpose 8a - 18-20 yr olds/CAT | Measure Name: PROMIS v1.0 - Meaning and Purpose Short Form 8a  Salsman JM, Lai JS, Hendrie HC, Butt Z, Zill N, Pilkonis PA, Peterson C, Stoney CM, Brouwers P, Cella D. Assessing psychological well-being: self-report instruments for the NIH Toolbox. Quality of Life Research. 2014 Feb;23:205-15. Salsman JM, Schalet BD, Park CL, George L, Steger MF, Hahn EA, Snyder MA, Cella D. Assessing meaning & purpose in life: development and validation of an item bank and short forms for the NIH PROMIS®. Quality of Life Research. 2020 Aug;29:2299-310. | © 2010-2023 PROMIS Health Organization (PHO) |
| PROMIS v1.0 - Meaning and Purpose 8a - Pediatric/CAT | Measure Name: PROMIS Pediatric v1.0 - Meaning and Purpose Short Form 8a Ravens-Sieberer U, Devine J, Bevans K, Riley AW, Moon J, Salsman JM, Forrest CB. Subjective well-being measures for children were developed within the PROMIS project: presentation of first results. Journal of clinical epidemiology. 2014 Feb 1;67(2):207-18. | © 2010-2023 PROMIS Health Organization (PHO) |
| PROMIS v1.0 - Psychological Stress Experiences 4a - Parent Proxy/CAT | Measure Name: PROMIS Parent Proxy v1.0 – Psychological Stress Experiences Short Form 4a Bevans KB, Gardner W, Pajer KA, Becker B, Carle A, Tucker CA, Forrest CB. Psychometric evaluation of the PROMIS® pediatric psychological and physical stress experiences measures. Journal of pediatric psychology. 2018 Jul 1;43(6):678-92. | © 2010-2023 PROMIS Health Organization (PHO) |
| PROMIS v1.0 - Psychological Stress Experiences 4a - Pediatric/CAT | Measure Name: PROMIS Pediatric v1.0 – Psychological Stress Experiences Short Form 4a Bevans KB, Gardner W, Pajer KA, Becker B, Carle A, Tucker CA, Forrest CB. Psychometric evaluation of the PROMIS® pediatric psychological and physical stress experiences measures. Journal of pediatric psychology. 2018 Jul 1;43(6):678-92. | © 2010-2023 PROMIS Health Organization (PHO) |
| PROMIS v1.0 - Self-Regulation - Flexibility 5a - Early Childhood Parent Report | Measure Name: PROMIS® Early Childhood Parent Report Scale v1.0 – Self-Regulation – Flexibility 5a  Blackwell CK, Kallen MA, Lai JS, Bevans KB, Wakschlag LS, Cella D. Measuring PROMIS® well-being in early childhood. Journal of pediatric psychology. 2022 Jun;47(5):559-72. | © 2010-2023 PROMIS Health Organization (PHO) |
| PROMIS v1.0 - Social Relationships - Family Relationships 4a - Early Childhood Parent Report | Measure Name: PROMIS® Early Childhood Parent Report Item Bank v1.0 – Social Relationships – Family Relationships – Short Form 4a Blackwell CK, Lai JS, Kallen M, Bevans KB, Davis MM, Wakschlag LS, Cella D. Measuring PROMIS® Social Relationships in Early Childhood. Journal of pediatric psychology. 2022 Jun;47(5):573-84. | © 2010-2023 PROMIS Health Organization (PHO) |
| PROMIS v1.0 - Social Relationships - Peer Relationships 4a -  Early Childhood Parent Report | Measure Name: PROMIS® Early Childhood Parent Report Item Bank v1.0 – Social Relationships – Peer Relationships – Short Form 4a Blackwell CK, Lai JS, Kallen M, Bevans KB, Davis MM, Wakschlag LS, Cella D. Measuring PROMIS® Social Relationships in Early Childhood. Journal of pediatric psychology. 2022 Jun;47(5):573-84. | © 2010-2023 PROMIS Health Organization (PHO) |
| PROMIS v1.2 - Global Health Scale | Measure Name: PROMIS v1.2 Scale - Global Health Hays RD, Bjorner JB, Revicki DA, Spritzer KL, Cella D. Development of physical and mental health summary scores from the patient-reported outcomes measurement information system (PROMIS) global items. Quality of life Research. 2009 Sep;18:873-80. | © 2010-2023 PROMIS Health Organization (PHO) |
| PROMIS v1.2 - Global Health Scale - 18-20 yr olds | Measure Name: PROMIS v1.2 Scale - Global Health Hays RD, Bjorner JB, Revicki DA, Spritzer KL, Cella D. Development of physical and mental health summary scores from the patient-reported outcomes measurement information system (PROMIS) global items. Quality of life Research. 2009 Sep;18:873-80. | © 2010-2023 PROMIS Health Organization (PHO) |
| PROMIS v2.0 - Anxiety 8a - Pediatric/CAT | Measure Name: PROMIS Parent Proxy v2.0 - Anxiety Short Form 8a Quinn H, Thissen D, Liu Y, Magnus B, Lai JS, Amtmann D, Varni JW, Gross HE, DeWalt DA. Using item response theory to enrich and expand the PROMIS® pediatric self report banks. Health and Quality of Life Outcomes. 2014 Dec;12:1-0. | © 2010-2023 PROMIS Health Organization (PHO) |
| PROMIS v2.0 - Companionship 6a - 18-20 yr olds | Measure Name: PROMIS v2.0 Companionship Short Form 6a Hahn EA, DeWalt DA, Bode RK, Garcia SF, DeVellis RF, Correia H, Cella D. New English and Spanish social health measures will facilitate evaluating health determinants. Health Psychology. 2014 May;33(5):490. | © 2010-2023 PROMIS Health Organization (PHO) |
| PROMIS v2.0 - Depressive Symptoms 8a - Pediatric/CAT | Measure Name: PROMIS Pediatric v2.0 - Depressive Symptoms Short Form 8a Quinn H, Thissen D, Liu Y, Magnus B, Lai JS, Amtmann D, Varni JW, Gross HE, DeWalt DA. Using item response theory to enrich and expand the PROMIS® pediatric self report banks. Health and Quality of Life Outcomes. 2014 Dec;12:1-0. | © 2010-2023 PROMIS Health Organization (PHO) |
| PROMIS v2.0 - Peer Relationships 7a - Parent Proxy/CAT | Measure Name: PROMIS® Parent Proxy Bank v2.0 – Peer Relationships – Short Form 7a Irwin DE, Gross HE, Stucky BD, Thissen D, DeWitt EM, Lai JS, Amtmann D, Khastou L, Varni JW, DeWalt DA. Development of six PROMIS pediatrics proxy-report item banks. Health and quality of life outcomes. 2012 Dec;10:1-3. | © 2010-2023 PROMIS Health Organization (PHO) |
| PROMIS v2.0 - Peer Relationships 8a - Pediatric/CAT | Measure Name: PROMIS Pediatric v2.0 - Peer Relationships Short Form 8a Irwin DE, Gross HE, Stucky BD, Thissen D, DeWitt EM, Lai JS, Amtmann D, Khastou L, Varni JW, DeWalt DA. Development of six PROMIS pediatrics proxy-report item banks. Health and quality of life outcomes. 2012 Dec;10:1-3. | © 2010-2023 PROMIS Health Organization (PHO) |
| Pubertal Development Scale | Petersen AC, Crockett L, Richards M, Boxer A. A self-report measure of pubertal status: reliability, validity, and initial norms. Journal of Youth and Adolescence. 1988:17(2);117-33. | Project on Human Development in Chicago Neighborhoods (PHDCN): Physical Development Scale, Wave 3, 2000-2002. |
| Rothbart Childhood Behavior Questionnaire Very Short Form | Rothbart MK, Ahadi SA, Hershey KL, Fisher P. Investigations of temperament at three to seven years: The Children's Behavior Questionnaire. Child development. 2001 Sep;72(5):1394-408. Putnam SP, Rothbart MK. Development of short and very short forms of the Children's Behavior Questionnaire. Journal of personality assessment. 2006 Jul 1;87(1):102-12. | Mary K. Rothbart |
| Rothbart Early Childhood Behavior Questionnaire Very Short Form | Putnam SP, Gartstein MA, Rothbart MK. Measurement of fine-grained aspects of toddler temperament: The Early Childhood Behavior Questionnaire. Infant behavior and development. 2006 Jul 1;29(3):386-401. Putnam SP, Jacobs J, Gartstein MA & Rothbart MK. (2010, March). Development and assessment of short and very short forms of the Early Childhood Behavior Questionnaire. Poster presented at International Conference on Infant Studies, Baltimore, MD. | Mary K. Rothbart |
| Rothbart Infant Behavior Questionnaire Revised Very Short Form | Putnam SP, Helbig AL, Gartstein MA, Rothbart MK, Leerkes E. Development and assessment of short and very short forms of the Infant Behavior Questionnaire–Revised. Journal of personality assessment. 2014 Jul 4;96(4):445-58. | Mary K. Rothbart |
| Sleep Health of Adults | Section B: Measure Name: PROMIS v1.0 – Sleep Disturbance – Short Form 4a Measure Name: PROMIS v1.0 – Sleep-related Impairment – Short Form 4a Buysse, D. J., Yu, L., Moul, D. E., Germain, A., Stover, A., Dodds, N. E., ... & Pilkonis, P. A. (2010). Development and validation of patient-reported outcome measures for sleep disturbance and sleep-related impairments. Sleep, 33(6), 781-792. Yu L, Buysse DJ, Germain A, Moul DE, Stover A, Dodds NE, Johnston KL, Pilkonis PA. Development of short forms from the PROMIS™ sleep disturbance and sleep-related impairment item banks. Behavioral sleep medicine. 2012 Jan 1;10(1):6-24. | © 2010-2023 PROMIS Health Organization (PHO) |
| Sleep Health of Adults - 18-20 yr olds | Section B: Measure Name: PROMIS v1.0 – Sleep Disturbance – Short Form 4a Measure Name: PROMIS v1.0 – Sleep-related Impairment – Short Form 4a Buysse, D. J., Yu, L., Moul, D. E., Germain, A., Stover, A., Dodds, N. E., ... & Pilkonis, P. A. (2010). Development and validation of patient-reported outcome measures for sleep disturbance and sleep-related impairments. Sleep, 33(6), 781-792. Yu L, Buysse DJ, Germain A, Moul DE, Stover A, Dodds NE, Johnston KL, Pilkonis PA. Development of short forms from the PROMIS™ sleep disturbance and sleep-related impairment item banks. Behavioral sleep medicine. 2012 Jan 1;10(1):6-24. | © 2010-2023 PROMIS Health Organization (PHO) |
| Sleep Health of Children and Adolescents - Child Self-Report | Section B:  Measure Name: PROMIS Pediatric v1.0 Sleep Disturbance Short Form 4a Measure Name: PROMIS Pediatric v1.0 Sleep-Related Impairment Short Form 4a Bevans KB, Meltzer LJ, De La Motte A, Kratchman A, Viél D, Forrest CB. Qualitative development and content validation of the PROMIS pediatric sleep health items. Behavioral sleep medicine. 2019 Sep 3;17(5):657-71. Forrest CB, Meltzer LJ, Marcus CL, De La Motte A, Kratchman A, Buysse DJ, Pilkonis PA, Becker BD, Bevans KB. Development and validation of the PROMIS Pediatric Sleep Disturbance and Sleep-Related Impairment item banks. Sleep. 2018 Jun;41(6):zsy054. | © 2010-2023 PROMIS Health Organization (PHO) |
| Sleep Health of Children and Adolescents - Parent Report | Section B:  Measure Name: PROMIS Early Childhood Parent Report v1.0 - Sleep Health Short Form 4b Lai JS, Blackwell CK, Tucker CA, Jensen SE, Cella D. Measuring PROMIS® physical activity and sleep problems in early childhood. Journal of pediatric psychology. 2022 Jun 1;47(5):534-46.  Section C: Measure Name: PROMIS Parent Proxy v1.0 Sleep Disturbance Short Form 4a  Measure Name: PROMIS Parent Proxy v1.0 Sleep-Related Impairments Short Form 4a Bevans KB, Meltzer LJ, De La Motte A, Kratchman A, Viél D, Forrest CB. Qualitative development and content validation of the PROMIS pediatric sleep health items. Behavioral sleep medicine. 2019 Sep 3;17(5):657-71. Forrest CB, Meltzer LJ, Marcus CL, De La Motte A, Kratchman A, Buysse DJ, Pilkonis PA, Becker BD, Bevans KB. Development and validation of the PROMIS Pediatric Sleep Disturbance and Sleep-Related Impairment item banks. Sleep. 2018 Jun;41(6):zsy054. | © 2010-2023 PROMIS Health Organization (PHO) |
| SRS-2 Preschool short form | Sturm A, Kuhfeld M, Kasari C, McCracken JT. Development and validation of an item response theory-based Social Responsiveness Scale short form. J Child Psychol Psychiatry. 2017 Sep;58(9):1053-1061. doi: 10.1111/jcpp.12731. | WPS, Licensed by the Measurement Core |
| SRS-2 School Age short form | Sturm A, Kuhfeld M, Kasari C, McCracken JT. Development and validation of an item response theory-based Social Responsiveness Scale short form. J Child Psychol Psychiatry. 2017 Sep;58(9):1053-1061. doi: 10.1111/jcpp.12731. | WPS, Licensed by the Measurement Core |
| The Everyday Discrimination Scale | Williams DR, Yu Y, Jackson JS, Anderson NB. Racial differences in physical and mental health: socioeconomic status, stress, and discrimination. Journal of Health Psychology. 1997;2(3):335-351. doi: 10.1177/135910539700200305. | Williams et al. 1997 |
